# Cryptic Patterning of Avian Skin Confers a Developmental Facility for Loss of Neck Feathering

**DOI:** 10.1371/journal.pbio.1001028

**Published:** 2011-03-15

**Authors:** Chunyan Mou, Frederique Pitel, David Gourichon, Florence Vignoles, Athanasia Tzika, Patricia Tato, Le Yu, Dave W. Burt, Bertrand Bed'hom, Michele Tixier-Boichard, Kevin J. Painter, Denis J. Headon

**Affiliations:** 1 The Roslin Institute and Royal (Dick) School of Veterinary Studies, University of Edinburgh, Edinburgh, United Kingdom; 2 UMR INRA/ENVT Laboratoire de Génétique Cellulaire, INRA, Castanet-Tolosan, France; 3 INRA, UE1295 PEAT, Nouzilly, France; 4 Laboratory of Natural and Artificial Evolution, Department of Zoology and Animal Biology, Sciences III, Geneva, Switzerland; 5 Facultad de Medicina, Universidad Nacional Autónoma de México, Mexico City, Mexico; 6 INRA, AgroParisTech, UMR1313 GABI, Jouy-en-Josas, France; 7 Department of Mathematics and Maxwell Institute for Mathematical Sciences, School of Mathematical and Computer Sciences, Heriot-Watt University, Edinburgh, United Kingdom; Stanford University School of Medicine, United States of America

## Abstract

Vertebrate skin is characterized by its patterned array of appendages, whether feathers, hairs, or scales. In avian skin the distribution of feathers occurs on two distinct spatial levels. Grouping of feathers within discrete tracts, with bare skin lying between the tracts, is termed the macropattern, while the smaller scale periodic spacing between individual feathers is referred to as the micropattern. The degree of integration between the patterning mechanisms that operate on these two scales during development and the mechanisms underlying the remarkable evolvability of skin macropatterns are unknown. A striking example of macropattern variation is the convergent loss of neck feathering in multiple species, a trait associated with heat tolerance in both wild and domestic birds. In chicken, a mutation called Naked neck is characterized by a reduction of body feathering and completely bare neck. Here we perform genetic fine mapping of the causative region and identify a large insertion associated with the Naked neck trait. A strong candidate gene in the critical interval, *BMP12*/*GDF7*, displays markedly elevated expression in Naked neck embryonic skin due to a cis-regulatory effect of the causative mutation. BMP family members inhibit embryonic feather formation by acting in a reaction-diffusion mechanism, and we find that selective production of retinoic acid by neck skin potentiates BMP signaling, making neck skin more sensitive than body skin to suppression of feather development. This selective production of retinoic acid by neck skin constitutes a cryptic pattern as its effects on feathering are not revealed until gross BMP levels are altered. This developmental modularity of neck and body skin allows simple quantitative changes in BMP levels to produce a sparsely feathered or bare neck while maintaining robust feather patterning on the body.

## Author Summary

The distribution of hairs or feathers across the body is not homogeneous, and many animals have characteristic regions of their skin with either profuse or reduced coverage. These features, such as manes, crests, or bald patches, are seen in diverse species, suggesting that they can be selectively advantageous and also that the mechanisms by which the skin develops somehow enables such features to appear repeatedly in the course of evolution. In this study we explore the basis of loss of neck feathering, a feature associated with heat tolerance that has arisen independently several times during bird evolution. We find that in chickens a bare neck is caused by increased production of BMPs, factors previously implicated in defining the size of the gaps between neighboring feathers. Selective production of retinoic acid by embryonic neck skin enhances BMP signaling, thereby bringing this skin region close to the threshold of BMP action required to completely suppress feather development. This usually innocuous distinction between neck and body skin enables mutations that increase BMP action to render the neck completely bare while permitting normal feathering on the body. Thus an underlying map within the skin provides a one-step route to a bare neck.

## Introduction

The vertebrate skin carries a highly ordered arrangement of pigments and morphological structures such as hairs and feathers. These patterns in the skin occur on two distinct spatial scales. Repetitive patterns of follicles or of pigment spots and stripes are laid out in a periodic manner, with each element in the micropattern positioned at a characteristic distance from its neighbors. On a larger anatomical scale, different parts of the body display periodic pattern variations in terms of the density and size of the repeated structures, and in regions of bare skin no periodic micropattern is present at all. These regional differences in micropattern across the skin constitute the macropattern.

Feathers are distributed in the avian skin on both of these spatial scales. The feather tracts, separated by bare skin, are macropattern elements, while the regular spacing between individual feathers defines the micropattern [1]–[3]. Both levels of organization arise in the embryo, beginning with the stereotypical positioning of the 14 feather tracts. In chicken this macropatterning is initiated at embryonic day 7 (E7) by dermal signals that induce stripes of cells that are competent to undergo feather development. These stripes can be detected using molecular markers to reveal the location of each incipient tract. The stripes broaden and propagate bilaterally across the skin, with micropatterning occurring just behind the propagating wavefront, resulting in the laying out of rows of feather primordia, called placodes [1],[4]. The placodes contain tightly packed cells that undergo rapid proliferation to produce a tubular outgrowth and subsequently undergo branching and differentiation to yield a mature feather fiber [5]–[7].

The sequential addition of new rows of feather placodes to tract margins terminates before the tracts meet, resulting in bare or downy spaces, called apterylae, between them. These bare patches persist through life and their area is associated with thermoregulatory capacity, particularly when present on the neck [8]–[10]. The extent and shape of feather tracts and apterylae are highly variable among bird species [11],[12], indicating an evolutionary malleability in the developmental processes that generate the skin's macropattern.

Distinct developmental mechanisms underlie the formation of macro- and microscale patterns. Classic embryological experiments have clearly demonstrated that of the two tissue layers that compose the skin, the dermis acts as the repository of positional information during macropatterning and that this information is conveyed to a positionally naïve epidermis [13]–[16]. In contrast to the rigid anatomical coordinates that define macropattern regions, experimental evidence and theoretical predictions [17]–[23] suggest that periodic micropatterning of the skin is achieved by the action of a reaction-diffusion mechanism whereby a field of cells is apportioned to placode or non-placode fates by the action of opposing Activatory and Inhibitory signals with specific regulatory connections and spatial ranges of action [24]–[26]. Such systems produce self-organizing patterns with relative pattern positions, in contrast to the absolute anatomical locations defined by the macropattern. The density of the pattern elements produced by these Activator-Inhibitor interactions depends on the relative potency of the Activator and the Inhibitor and their spatial ranges of action. In studies of micropatterning of chicken and mouse skin, experimental evidence points to members of the BMP family as being Inhibitory factors [17],[18],[21],[27], while WNT/β-catenin [20],[28]–[32] and FGF [33]–[36] pathways act as Activators.

Standard reaction-diffusion systems yield a single characteristic follicle density as the pattern output [25],[37]. However, the skin's micropattern is not uniform across the entire body, raising the question of how different densities of hair and feather follicles, or patches of entirely bare skin, are laid out to achieve the diverse skin patterns so characteristic of the external anatomy of the vertebrates. Here we address this question by analyzing the genetic and developmental basis of the Naked neck chicken, an example of macropattern variation in a single species.

In order to dissect the mechanisms that modulate feather patterning on neck skin, we had previously mapped the Naked neck (*Na*) mutation to a 13 cM interval in the distal region of chicken chromosome 3q, 5.7 cM from the closest microsatellite marker [38]. Here we use the original mapping family to fine-map the causative gene by searching for recombination breakpoints with SNP (Single Nucleotide Polymorphism) markers in order to narrow the interval. By analyzing the candidate genes in the reduced interval, we found that the Naked neck mutation causes elevated expression of the *BMP12* gene in developing skin, which is associated with a large insertion approximately 260 kb from *BMP12*. We then establish that the feather patterning of the neck skin is influenced by the existence of a cryptic molecular macropattern that has modest phenotypic effects until revealed by alteration of BMP levels. This illustrates how the periodicity-generating interactions of a reaction-diffusion network are integrated with the positional information encoded at different anatomical sites to produce the skin's diverse macropattern.

## Results

### The Basis of the Naked Neck Trait in Domestic Fowl

**Figure 1:**
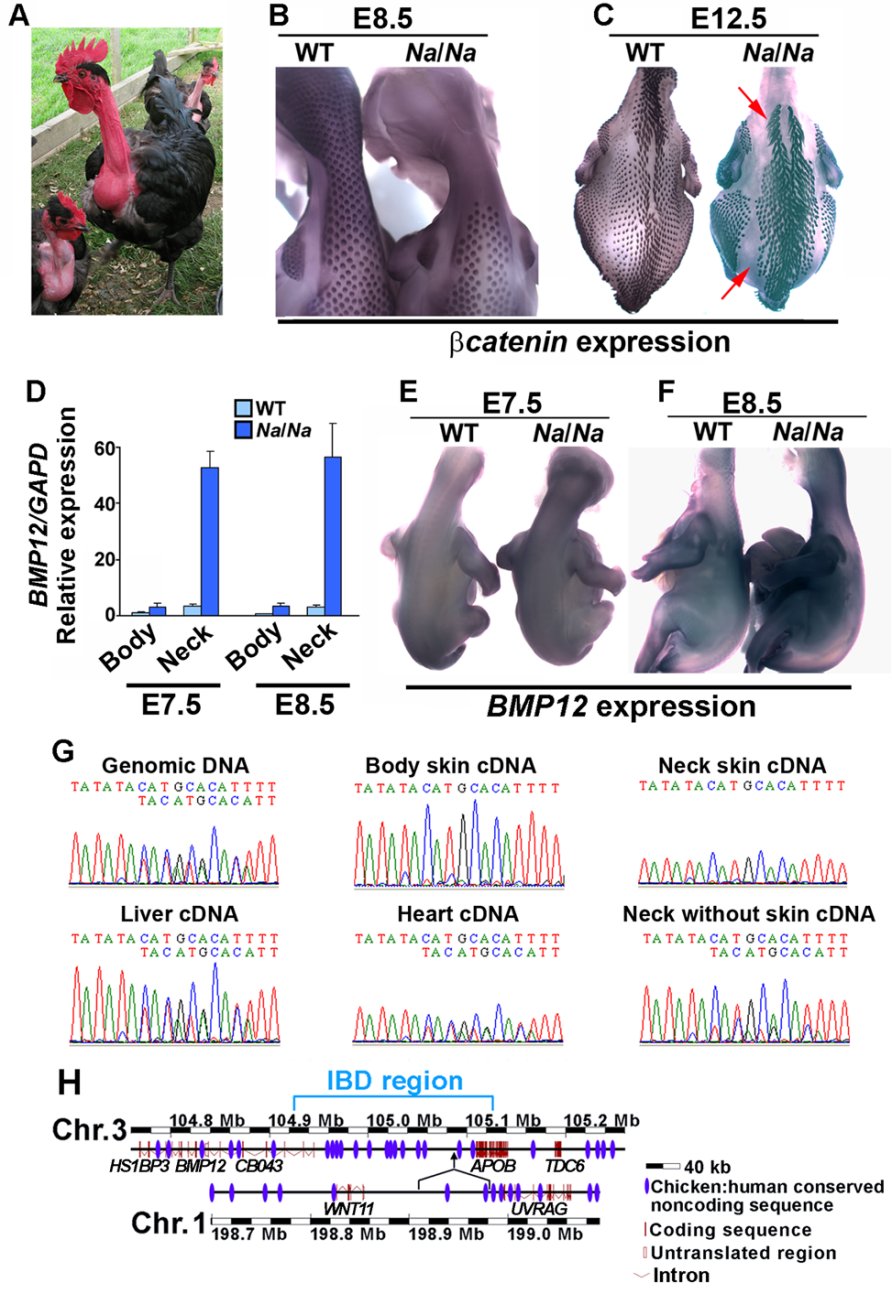
The Naked neck phenotype is caused by a cis-regulatory mutation that results in elevated *BMP12* expression. (A) Adult *Na*/*Na*. Feathers are absent on the neck and head, excepting the crown. (B) E8.5 embryos hybridized with a *β*-*catenin* probe to mark the patterning field and feather primordia. Punctate expression of *β*-*catenin* in feather placodes is seen on the body but not the neck of the mutant. WT, wild type; *Na/Na*, Naked neck. (C) E12.5 embryos showing limited lateral tract expansion (arrows) in *Na*/*Na*, reducing body feather coverage. (D) Quantitative RT-PCR determination of *BMP12* expression in body and neck skin of E7.5 and E8.5 wild type and *Na*/*Na* embryos. (E,F) In situ hybridization detecting *BMP12* in wild type and *Na/Na* embryos at (E) E7.5 and (F) E8.5. Wild type and mutant embryos were hybridized and photographed together. *Na/Na* embryos have elevated and diffuse expression of *BMP12* in the skin. (G) Sequence traces of PCR products from E8.5 *Na/+*. Genomic DNA PCR products display double peaks following a TA indel polymorphism in the *BMP12* 3′UTR. RT-PCR products from neck and body skin show a single trace throughout, indicating predominant expression of the Naked neck *BMP12* allele, while both alleles are detected in RT-PCR products from other tissues. (H) Schematic showing insertion of chromosome 1 sequences into chromosome 3 at the Naked neck locus. Chromosome coordinates, the Naked neck identical by descent segment, gene names, exons, untranslated regions, and non-coding elements conserved between chicken and human genomes, based on the ENSEMBL genome viewer, are indicated.

Domestic Naked neck fowl lack feathers on the neck and have narrow feather tracts on the body (Figure 1A). As in wild species, the Naked neck trait in chicken is associated with enhanced thermotolerance and with increased agricultural production in hot climates [39],[40]. This trait is caused by a single incompletely dominant locus, which abolishes neck feathering and reduces body feathering by approximately 20% in heterozygotes and by 40% in homozygotes [41]. Patterning of feathers, rather than their morphogenesis or maintenance, is affected by the Naked neck mutation as mutant embryos lack feather placodes on the neck and display reduced tract expansion on the body (Figure 1B and 1C). Naked neck embryos and adults exhibit a discrete boundary between feathered and unfeathered regions, though in wild type birds there is no overt boundary demarcating neck from body skin (Figure 1B and 1C) and both regions are considered to carry a continuous spinal tract that runs from head to tail [11],[12].

To gain molecular insight into the basis of macropattern variations, we started by refining the location of the causative mutation. As we had already mapped the *Na* locus to a 13 cM interval of chicken chromosome 3 [38], we developed 11 new markers from this region to refine the location in the original mapping family. Recombination events in two individuals led to refinement of the candidate gene to a region of 770 kb, containing five annotated genes (Figure S1). We sequenced all predicted exons of these genes (*HS1BP3*, XM_419977; *BMP12*, XR_026709; *CB043*, NM_001031093; *APOB*, NM_001044633; and *TDC6*, XM_419980) from *Na/Na* genomic DNA and did not identify any mutations predicted to affect the coding sequences or splice junctions of any of these genes. This suggested that the *Na* mutation influences transcriptional regulation, resulting in altered expression of one or more genes in the region. We found that only one of the five candidate genes, *BMP12* (also known as *GDF7*), is normally expressed in developing skin and embryonic feather placodes (Figure S2), and that this gene exhibits strongly increased expression in Naked neck mutant skin at the onset of feather patterning (Figure 1D). None of the other genes within the *Na* critical region has altered expression levels in Naked neck mutant skin (Figure S3). In situ hybridization revealed that the elevated expression is widespread throughout the skin of mutant embryos (Figure 1E and 1F). By sequencing across an indel polymorphism in the 3′UTR of *BMP12*, we found that in *Na/+* heterozygous embryos the expression of the mutant allele is greater than that of the wild type in the skin, but not in internal organs (Figure 1G), demonstrating the action of a cis-regulatory mutation with a tissue-specific effect.

To further refine the location of the genetic modification causing the Naked neck trait, we genotyped multiple wild type and Naked neck individuals from geographically dispersed flocks for markers across the 770 kb critical region. This identified an approximately 200 kb region that was identical by descent in all available *Na/Na* individuals (Table S1). While tiling this region by overlapping PCRs we found that we could not amplify across one specific region (chromosome 3: nucleotides 105089664–105089844) in Naked neck individuals, suggesting the presence of a genomic rearrangement at this location. We used inverse PCR to define the sequences flanking this rearrangement, finding on both sides the insertion of chromosome 1 sequences that map 73 kb apart from one another in the reference genome (Figure 1H, Figure S4). These inserted sequences map to an intergenic region flanked by the *WNT11* (NM_204784) and *UVRAG* (NM_001030839) genes on chromosome 1. We confirmed the presence of a large insertion at this location by PCR using chromosome 1 and chromosome 3 primers (Figure S5) and further confirmed that this insertion was both present in all Naked neck genomes available and absent from >500 wild type chromosomes from diverse breeds (Table S2). As this large insertion is unique to Naked neck genomes it appears that this mutation is responsible for the increased *BMP12* expression in skin of Naked neck embryos through a long-range (>260 kb downstream) cis-regulatory effect.

### Elevated BMP Signaling Causes the Naked Neck Trait

**Figure 2:**
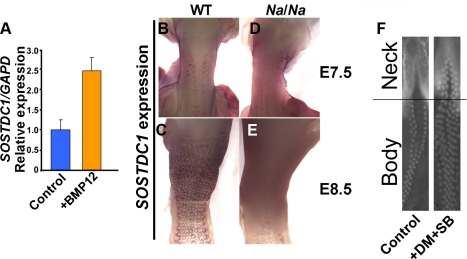
Naked neck skin displays elevated BMP signaling. (A) Application of recombinant BMP12 to cultured skin for 15 h leads to elevation of *SOSTDC1* expression, determined by quantitative RT-PCR. (B–E) Detection of *SOSTDC1* expression by in situ hybridization. (B) At E7.5 wild type embryos have two rows of feather placodes running up the neck. *SOSTDC1* is expressed at the periphery of the placodes and is not detected in the medial region between the lateral rows of placodes. (C) By E8.5 the medial region of the neck is populated by feather placodes. (D) E7.5 *Na/Na* embryos have placodes on the dorsum, but widespread *SOSTDC1* expression on the neck, including the medial region. (E) At E8.5 the Naked neck skin maintains a high level of widespread *SOSTDC1* expression, with peri-placode expression visible on the body. (F) Ex vivo rescue of the Naked neck phenotype by suppression of BMP signaling. E7.0 *Na/Na* skin was cultured in the presence of dorsomorphin (DM, used at 8 µM) and SB203580 (SB, used at 5 µM), pharmacological inhibitors of BMP signal transduction, for 48 h. This permitted feather development across most of the mutant neck skin.

As several BMP family members act during early feather development [17],[18],[42],[43] we determined whether the increased *BMP12* expression in Naked neck embryos leads to an appreciably increased overall BMP signal response. *SOSTDC1* (NM_204373) is a target of BMP signaling in developing mouse skin [21] and we confirmed that this gene is a BMP target in chicken skin also (Figure 2A). We then used *SOSTDC1* as a marker to visualize the distribution of BMP responses in the developing neck skin. *SOSTDC1* expression is detected at the periphery of nascent feather placodes in wild type skin (Figure 2B), consistent with these zones experiencing BMP-mediated lateral inhibition of feather identity during periodic patterning. At E7.5 the anterior region of the spinal tract, including the neck, displays one row of feather primordia on each side of the midline, and over the next 24 h the entire dorsal region of the neck becomes populated with feather placodes (Figure 2C). In contrast, Naked neck embryos display a broad swathe of *SOSTDC1* expression across the neck (Figure 2D and 2E), consistent with the failure of feather placode formation in this region being a result of inhibition by elevated BMP12 levels. Confirming that excessive BMP signaling causes the Naked neck phenotype, we found that pharmacological suppression of BMP signal transduction rescues feather development on the neck of cultured *Na/Na* skin (Figure 2F).

### BMP Sensitivity Is Greater in Neck than in Body Skin

**Figure 3:**
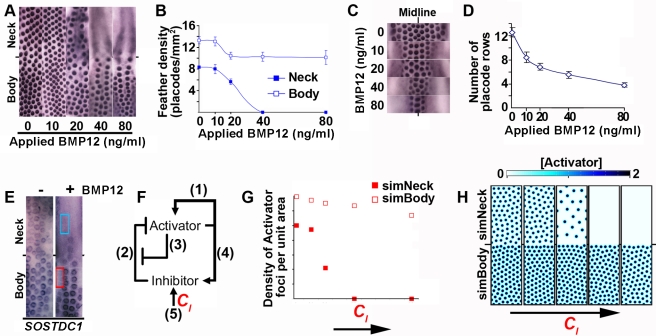
Differential sensitivity to BMP signals alters neck patterning while maintaining body feather placode periodicity and size. (A,B) *β*-*catenin* in situ hybridization revealing the effects of recombinant BMP12 application on feather periodicity and regional distribution in wild type skin after 48 h. (C,D) Dose effects of BMP12 on the number of feather placode rows on the spinal tract of the body. Feather primordia are visualized by *β*-*catenin* in situ hybridization. (E) *SOSTDC1* expression on control and 80 ng/ml BMP12 treated skin explants. Feather placodes express *SOSTDC1* at their periphery on both body and neck. Upon application of BMP12, the non-placode skin of the neck expresses a higher level of *SOSTDC1* than does the body (compare signal intensity in the red boxed area to that of the blue boxed area). (F) Schematic of reaction-diffusion regulatory interactions. Adjacent numbering refers to mathematical terms in the supporting methods. *C_I_* represents the constitutive, ubiquitous production of the Inhibitor. (G) Quantification of periodicity of Activator foci in simulated neck and body with differential sensitivities to Inhibitor. *C_I_* increases along the *x*-axis. (H) Pattern outcomes from reaction-diffusion dynamics in a field with graded sensitivity to the Inhibitor. Abolition of Activator foci in the more sensitive part of the field is achieved with little effect on periodic spacing in the remainder of the field, producing a macropatttern that matches the effects of BMP12 treatment on cultured skin. Colors denote local Activator concentrations, with black representing the highest and white the lowest Activator levels. Areas with high Activator concentration represent placodes.

Initially, we considered that the basis for the complete loss of neck feathers coupled with retention of body feathers in Naked neck mutants was likely a result of the disproportionate elevation of *BMP12* expression in *Na/Na* neck skin compared to body skin (Figure 1D). However, we found that treating explant cultures of wild type skin with soluble BMP12 protein did not cause a homogeneous disruption of feather patterning, but instead reproduced the Naked neck phenotype (Figure 3A and Figure S6). Application of recombinant BMP4 yielded similar results, demonstrating that this skin regional effect on feather placode suppression is not a unique property of BMP12 but is general to these BMP ligands. Although the strongly elevated *BMP12* expression on neck compared to body skin in chickens carrying the *Na* mutation is likely to influence the precise nature of the feather macropattern in this mutant, the greater sensitivity to BMP signals of the neck relative to the body in wild type embryos is sufficient to enable loss of neck feathering in response to quantitative changes in total BMP levels.

This finding demonstrates that regional macropatterning of avian skin, in particular the distinction between the neck and body, involves the same signaling molecules as employed for the conceptually distinct periodic micropatterning of individual feathers. To explore the relationship between periodic and anatomical patterning, we treated skin with different doses of BMP12 and assessed the effects on feather density and on body tract width. We found that the density of feather placodes on the neck is normally lower than that of the body and that neck skin placode density falls sharply when exposed to exogenous BMP12. In contrast, on the body the periodic pattern is relatively robust to increasing BMP12 levels (Figure 3A and 3B), though this treatment causes a dose-dependent reduction in the number of placode rows, and hence overall tract size (Figure 3C and 3D). To visualize regional differences in BMP-sensitivity we assessed *SOSTDC1* expression in response to applied BMP12 and found elevated BMP responses on the neck, with a sharp gradient of sensitivity from neck to body (Figure 3E). Thus BMPs elicit greater transcriptional responses on the neck, in addition to being more effective inhibitors of feather development in this region.

### Distinct BMP Thresholds Permissive for Micropatterning on Neck Versus Body Skin

The periodic micropatterning of feather placodes relies on the interaction of factors that activate or inhibit placode formation [17],[18],[42],[44], operating in a reaction-diffusion mechanism. BMPs have been proposed to represent inhibitory factors during feather placode patterning [17],[18], with the WNT/β-catenin and FGF pathways serving as key activators. Reaction-diffusion mechanisms rely on the action of an Activator, which stimulates production of more Activator in a positive feedback loop and which also promotes the synthesis of its own Inhibitor. Attainment of a high Activator concentration by a cell alters its fate, in this case to that of feather placode. When the Inhibitor possesses a greater range of action than the Activator and when the relative signaling potencies of Activator and Inhibitor are appropriately balanced, these interactions will produce a periodic pattern from near homogeneous initial conditions [24]–[26]. In such systems Inhibitor production is a result of both widespread, constitutive synthesis starting prior to patterning, denoted here by *C_I_*, as well as the Activator-induced Inhibitor upregulation that occurs during the patterning process (Figure 3F). We performed computational simulations to determine whether the operation of a reaction-diffusion system on a field with differing Inhibitor sensitivities could explain the different neck versus body patterning behaviors observed upon BMP12 treatment of embryonic skin. We applied differential Inhibitor sensitivity to our patterning field according to the profile of *SOSTDC1* expression in BMP12 stimulated skin. Thus the simulations now explored periodic patterning on a field with an Inhibitor sensitive region, representing the neck, and a less sensitive region, representing the body, with a steep gradient of Inhibitor sensitivity between these regions. Varying *C_I_* in the patterning simulations, which mimics the application of recombinant BMP12 to cultured skin, altered the simulated placode patterns in the manner observed in experimental treatments. Thus, high *C_I_* values caused ablation of Activator foci in the sensitive “neck” domain, while pattern density on the simulated body was little affected (Figure 3G and 3H). As observed in Naked neck fowl and in BMP12 treated skin cultures, the simulations also yielded a sharp boundary between the neck and body, the location of which was stable with varying Inhibitor levels (Figure 3H). Further simulations testing a range of Inhibitor sensitivity gradient slopes revealed that the observed sharp, but not step-change, gradient of Inhibitor sensitivity best fits our experimental observations of chicken skin pattern behavior (Figure S7).

**Figure 4:**
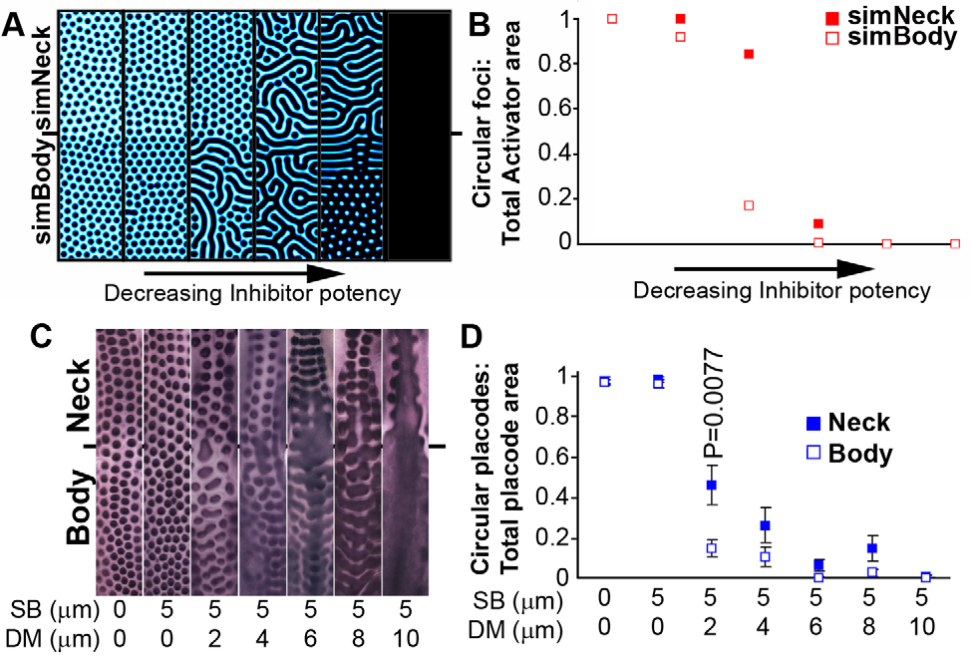
Regional disparity in pattern behavior upon suppression of BMP signal transduction. (A) Simulated pattern outcomes upon reduction of Inhibitor potency. Transition from production of circular foci to a striped pattern occurs, first on the less sensitive (simulated body) region, followed by stripe formation on the more sensitive domain (simulated neck) at higher levels of signal suppression. (B) Quantification of pattern characteristics from simulation of diminished Inhibitor potency. The proportion of total Activator positive area that is represented by circular foci is plotted. (C) *β*-*catenin* in situ hybridization detecting placode pattern upon suppression of BMP signal transduction in cultured E7.0 chicken skin. SB, p38 MAPK inhibitor SB203580; DM, Smad1/5/8 inhibitor dorsomorphin. Inhibition of p38 MAPK has little effect on the placode pattern, but yielded a robust effect in concert with suppression of Smad function. At low doses of DM stripes begin to form first on the body, then at higher doses on the neck. High doses cause *β*-*catenin* expression throughout the skin. (D) Quantification of the proportion of total *β*-*catenin* positive area that is represented by circular placodes in cultured skin treated with BMP inhibitors. Statistically significant *p* values are indicated above data points.

In contrast to the effects of augmenting Inhibitor production, our simulations predicted that graded suppression of BMP function would produce stronger pattern alterations on the body than on the neck. A numerical sensitivity analysis of the model demonstrated that moderate suppression of the Inhibitor causes a transition from a spotted pattern to a striped one (Tables S3 and S4; Figure S8) [45],[46] and our model predicted that such spot to stripe transitions would occur readily on the body, with stripe production on the more sensitive neck requiring further suppression of the Inhibitor's action (Figure 4A and 4B). We tested this prediction by inhibiting the Smad1/5/8 and p38MAPK transducers of the bifurcated BMP signaling pathway [47] in cultured skin. We found, as predicted by simulation, that neck and body patterns did indeed respond differently to BMP signal suppression, with stripes being more prevalent on body than neck skin at low doses, while upon further suppression of BMP responses the pattern on body and neck converged to yield ubiquitous *β*-*catenin* expression within the tracts (Figure 4C and 4D). Intuitively, this phenomenon can be understood as the suppression of Inhibitor/BMP activity leading to over-accumulation and saturation of the opposing Activator levels, and hence to expansion of Activator foci. The symmetric expansion of Activator foci becomes restricted with the narrowing of the inhibited zones separating them and adjacent foci are thus forced to expand laterally, creating elongated placodes. As more lateral expansion of foci occurs, the prevailing pattern becomes one of activated stripes, rather than spots. The higher sensitivity of neck skin prevents Activator over-accumulation at moderate levels of Inhibitor/BMP suppression, requiring further suppression of BMP signaling to achieve Activator saturation and stripe production. These findings show that a reaction-diffusion system operating on a field with different Inhibitor sensitivities explains both the modest difference in placode density between neck and body in unmanipulated embryonic skin, as well as the greater pattern divergences between neck and body caused by experimental titration of BMP signaling.

### Retinoic Acid Sensitizes Developing Skin to Placode Suppression by BMP Signaling

**Figure 5:**
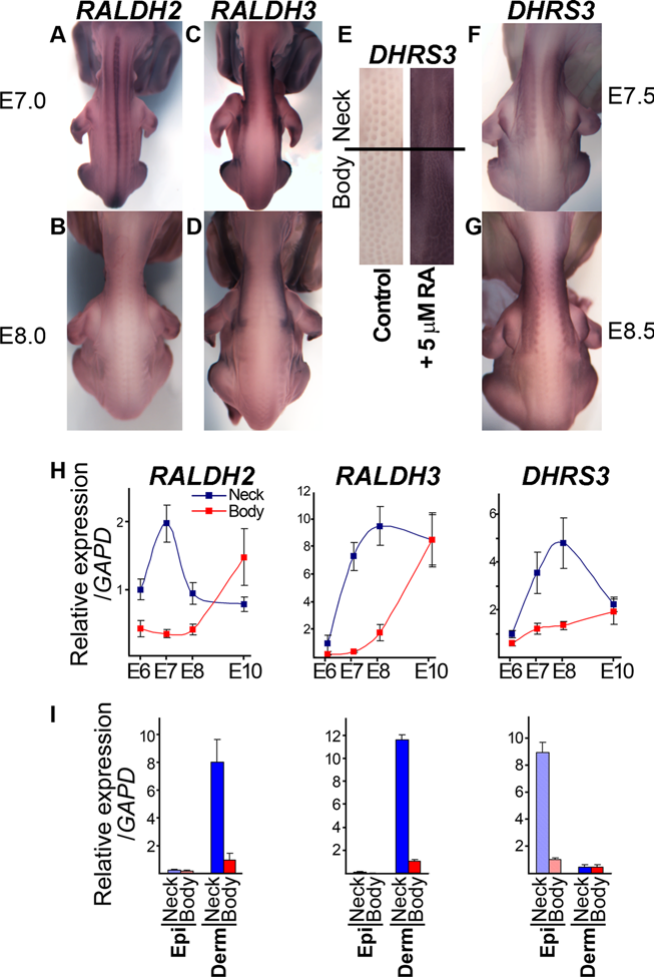
Retinoic acid production and signaling in neck skin distinguishes this region from the body. (A,B) Detection of *RALDH2* expression in E7.0 and E8.0 embryos by whole mount in situ hybridization. *RALDH2* is expressed more strongly in neck skin than in body skin and is also detected in the neural tube (midline). (C,D) *RALDH3* is expressed broadly in neck skin at E7.0 and moves laterally by E8.0. (E) Expression of the RA target gene *DHRS3* in skin cultured from E7.0 for 2 d in the presence or absence of 5 µM RA. Both neck and body skin respond to RA. (F,G) In vivo *DHRS3* is expressed on the neck, but not the feather tract of the body. (H) Quantitative RT-PCR detecting *RALDH2*, *RALDH3*, and *DHRS3* expression in neck and body skin from E6 to E10. The disparity between neck and body skin is greatest at E7 and E8, when feather patterning is taking place. *DHRS3* levels track *RALDH2* expression dynamics more closely than those of *RALDH3*. (I) Quantitative RT-PCR detection of *RALDH2*, *RALDH3*, and *DHRS3* expression in separated epidermis (Epi) and dermis (Derm) at E7.0. The RA producing enzymes are expressed in the dermis, while RA target gene expression is activated in the epidermis.

To elucidate the molecular basis of the different sensitivities of chicken neck and body to BMPs, we compared the gene expression profiles of these two skin regions by array hybridization at E7.0 (Table S5). This approach identified expression of components of the retinoic acid (RA) signaling pathway as a very prominent difference between neck and body skin. The RA synthesizing enzymes *RALDH2* (NM_204995) and *RALDH3* (NM_204669) [48],[49] and the RA target genes *DHRS3* (XM_417636) and *CYP26A1* (NM_001001129) [50],[51], displayed significantly elevated expression in neck compared to body skin. RA signaling is important for determining skin appendage identity and orientation during morphogenesis [52],[53] but has not previously been implicated in influencing the periodic patterning of skin appendages. Whole mount in situ hybridization confirmed that *RALDH2* expression is more pronounced on neck than body, with strong expression also observed in developing neural tissue along the midline (Figure 5A and 5B). *RALDH3* expression was predominantly on the neck, with some extension onto the body peripheral to the presumptive feather tract (Figure 5C and 5D), and prominent expression on the hindlimb at the margin of the femoral tract was also observed (Figure S9). Visualization of the sites of RA signal responses by detection of *DHRS3* showed that while neck and body skin are both sensitive to the action of exogenous RA (Figure 5E), endogenous RA synthesis elicits responses specifically on the neck and in a diminishing gradient onto the anterior region of the body at the lateral margins of the feather tract (Figure 5F and 5G). The definition of the neck as a site of selective RA signaling is not unique to chicken as we observed very similar RA pathway gene expression profiles in duck, turkey, quail, and guinea fowl embryos during their feather patterning (Figure S10). Quantification of RA pathway gene expression revealed the transient nature of the neck/body disparity, with the neck displaying higher transcript levels only during feather patterning (E7 and E8) and little difference between neck and body prior to and following completion of this process (Figure 5H). To determine which skin layer produces RA and which layer responds to this signal, we quantified gene expression in isolated dermis and epidermis (Figure 5I). *RALDH2* and *RALDH3* expression were detected only in the dermis, while *DHRS3* expression was predominantly epidermal. This shows that RA is produced in the dermis and acts as a signal to the overlying epidermis, a finding consistent with classical skin recombinations which demonstrated that macropattern information is encoded within the dermis [13].

**Figure 6:**
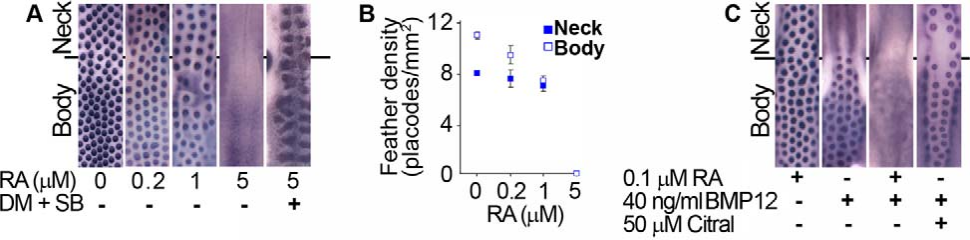
Retinoic acid potentiates BMP inhibition of feather patterning. (A) RA administration reduces the density of placodes, which are detected by *β*-*catenin* in situ hybridization, completely inhibiting placode formation at high doses. Suppression of BMP signaling with 4 µM dorsomorphin and 5 µM SB203580 rescues placode formation in the presence of RA. (B) Quantification of placode density on neck and body upon RA treatment. With increasing doses of RA the feather density on body and neck converges and ultimately all feather placode formation is suppressed. (C) RA sensitizes body skin to BMP-driven inhibition of feather development. The application of 0.1 µM RA has little effect on the placode pattern and application of 40 ng/ml BMP12 permits placode formation on the body. Co-treatment with RA and BMP12 has a synergistic effect, completely suppressing feather development on the body. Conversely, treatment of skin with the RA synthesis inhibitor Citral renders the neck resistant to suppression of placode formation by BMPs.

Based on the finding that RA signaling occurs at higher levels in neck than body skin at the onset of feather patterning, we considered that this factor might be responsible for the heightened sensitivity of neck skin to BMP-mediated inhibition of feather development. We tested this first by determining the effect of RA on placode patterning, and then by asking whether the differences in patterning behavior observed between neck and body skin could be minimized by reducing the difference in RA signal intensity between these two regions. We found that RA acts as an inhibitor of feather placode formation, with increasing doses of RA leading to a reduction in placode density and ultimately to complete suppression of placode formation (Figure 6A and 6B). In contrast to BMP administration, RA signaling effectively suppresses placode formation on both neck and body. RA inhibition of placode formation requires active BMP signaling (Figure 6A), suggesting that the primary action of RA might be to sensitize the skin to BMP signals. To test this idea directly, we co-treated skin with modest doses of both RA and BMP12 and observed a synergistic effect of these two signals, with low doses of RA potentiating the action of BMPs to allow complete suppression of placode formation on the body (Figure 6C). Thus the ability of the body skin to resist BMP signals, which enables feather development in the presence of moderate levels of BMP, depends on the absence of RA signaling in this region. To confirm that RA signaling is responsible for sensitizing the neck to BMP action, we cotreated skin cultures with Citral, an inhibitor of the RALDH enzymes, together with BMP12 and found that this suppression of endogenous RA production allowed feather patterning on the neck (Figure 6C). These results show that RA sensitization of skin to BMP signals accounts for the different pattern behaviors of neck and body skin, allowing quantitative changes in gross BMP levels to selectively reduce or abolish neck feathering.

## Discussion

Hairs and feathers are laid out in different patterns on different parts of the body according to their roles in thermoregulation, defense, and display. We have explored the developmental basis for variation of neck feathering in birds, finding that the Naked neck trait in domestic fowl is caused by suppression of embryonic feather development through increased *BMP12*/*GDF7* expression. This adds to the catalog of agricultural production traits associated with altered GDF (Growth and Differentiation Factor) function, which includes increased muscle growth for meat production (*GDF8*/*myostatin*) [54],[55] and fecundity (*GDF9B*/*BMP15*) [56] in livestock.

The increased *BMP12* expression that we observe in *Na* skin is completely associated with the insertion of chromosome 1 sequence downstream of this gene. This inserted sequence lies between *WNT11* and *UVRAG* and contains conserved non-coding elements, but no sequence predicted to be transcribed. While determination of the precise mechanism of action of the mutation requires further investigation, the well-characterized expression of *WNT11* in developing chicken skin [57] suggests that *BMP12* expression may be upregulated in *Na* mutants due to the acquisition of *WNT11* enhancers lying within the insertion. This notion is supported by our finding that upregulation of *BMP12* in Naked neck embryos is particularly strong on the neck compared to the body (Figure 1), and *WNT11* expression also appears to be significantly stronger on the neck than the body (Table S5). Alternatively, the insertion could act to abolish the function of a repressive regulatory element on chromosome 3, a similar mechanism having been shown to be the cause of increased *IGF2* expression contributing to enhanced muscle growth in pigs [58],[59]. The large distance between the insertion and the *BMP12* coding sequence that it influences is consistent with an emerging picture of the strikingly long-range action of cis-regulatory elements that tend to be responsible for control expression of BMP family genes [60].

Based on their expression patterns and ability to suppress feather development, BMP family members have been proposed to be Inhibitors in a reaction-diffusion system that dictates the micropattern spacing between individual feather follicles [17],[18],[42], though no genetic evidence in favor of such an activity in vivo has previously been reported. Using graded stimulation and suppression of BMP signaling coupled with analysis of pattern transitions, we provide further evidence in support of the BMP family playing the key Inhibitory roles during periodic feather patterning. More importantly, we find that different regions of the skin display differing sensitivities to BMPs during feather patterning, revealing a molecular link between micro- and macroscale patterning. Appropriately balanced activities of Activatory and Inhibitory signals are key to the operation of reaction-diffusion systems; if either function is too potent, then no periodic pattern can be produced. Thus, above a given threshold of BMP signaling, the micropattern Activatory functions (probably mediated by WNTs and FGFs [28],[29],[33]–[36], though the precise regulatory connections between BMPs and these genes remain to be defined) are overwhelmed and cannot stabilize the positive feedback loop required to generate placodes. In this way a region of skin can be rendered refractory to periodic patterning by the amount of BMP signaling it experiences.

**Figure 7:**
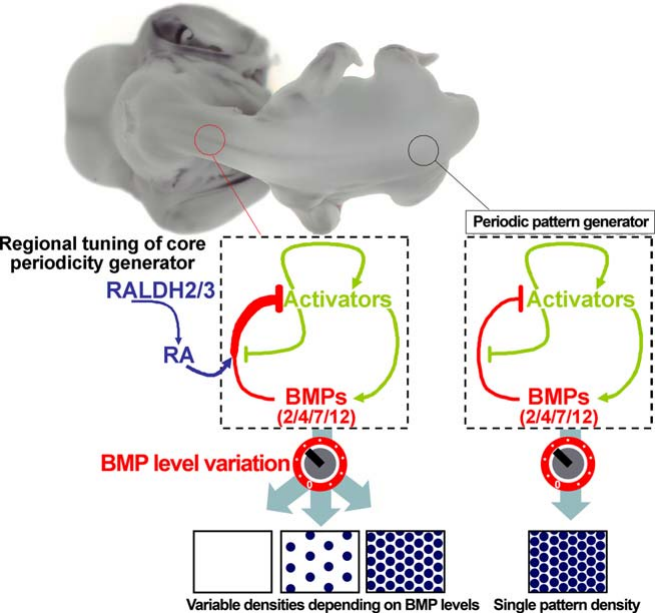
Schematic of periodic pattern formation neck and body skin. A single core periodic patterning system based on a reaction-diffusion mechanism operates across the body and neck. Such a system operating in isolation has a single characteristic wavelength, thus producing placodes at a single density (right). Sensitization of neck skin to BMP signals as a result of RA production in this region alters the output of the patterning mechanism, allowing a reduction in feather density or the abolition of neck feathering, depending on the global level of BMP at the onset of patterning.

That neck skin has a greater sensitivity to BMP signals than body skin demonstrates that the apparently continuous spinal feather tract is in fact composed of two partly independent developmental modules. This modularity is enabled by the level of RA signaling, which is high on the neck and low on the body due to differential expression of *RALDH* genes. RA plays a key role in defining the placode pattern on neck skin by potentiating BMP signaling, thereby reducing feather density in a manner that depends on gross BMP levels. It is important to note that RA does not itself act as a component of the periodicity generator as we observe no evidence that RA synthesis within feather placodes acts to laterally inhibit placode identity in surrounding skin (Figure 5). Rather, RA acts as an external input that modulates the output of the periodic patterning mechanism (Figure 7). Previous theoretical studies have indicated that spatially distributed inputs can significantly modulate the form and variety of patterning [61]–[63] and the results here suggest that this type of external modulation is likely to be a recurring theme in reaction-diffusion patterning, as the imposition of such inputs allows a single set of Activator-Inhibitor interactions to produce distinct pattern outputs on different regions of a field, yielding a macropattern.

As no new feathers are inserted between existing ones as the skin expands to maturity, the adult feather pattern is a product of both the cell signaling processes focused on here together with the diluting effects of subsequent skin growth. Though placode density on the embryonic neck is significantly lower than that of the body, in adults the neck and body feather densities are the same (Figure S11). Thus the impact of RA in reducing neck placode density during patterning is compensated for by subsequent unequal growth of neck and body skin, with the body pattern being stretched to a greater degree than the neck, ultimately resulting in a homogeneous feather distribution across these two regions in the adult.

We also find that the lateral parts of the body skin are more sensitive to BMP-mediated suppression of feather development than the medial skin. However, we see no evidence that RA is involved in this phenomenon, as RA pathway genes are not expressed by lateral body skin and suppression of RA production using Citral does not impair BMP-driven reduction in body tract width (Figure 5 and Figure 6C). It is likely that this apparent medial-lateral BMP sensitivity gradient simply reflects the later formation of placodes on lateral than on medial skin. This results in lateral skin experiencing a greater duration of BMP stimulation prior to placode formation than medial skin. In addition, ventral skin is also likely to exhibit a higher BMP sensitivity than dorsal skin, since a marked reduction of feather cover is observed on the belly region of homozygous *Na/Na* individuals, while *Na/+* heterozygotes have a more normally feathered ventrum. Thus different BMP sensitivities, perhaps based on a range of different molecular mechanisms, may play a widespread role in defining the macropattern across the entire body.

These findings have implications for the developmental mechanisms underlying the evolutionary diversity of skin patterns. During the course of avian evolution neck feathering has been lost independently in several lineages, notably in large species of the tropics, such as members of the Accipitridae (Old World vultures), Cathartidae (New World vultures), Ciconiidae (genus *Leptoptilos*, including the Marabou stork), and the large ratites (ostrich, emu, cassowary, and rhea). The fossil record does not support a bare neck as an ancestral feature of the feather pattern [64], raising the question of how this character could have evolved so frequently. The modularity of neck skin that we report illustrates that the positional information distinguishing neck from body is generally present in avian embryonic skin, requiring only changes to gross signal levels to produce a sparsely feathered or bare neck. In general, developmental modularity of this kind enhances evolvability by dissociating the effects of genetic change on distinct anatomical regions [65],[66]. The presence of cryptic skin patterns removes the need for evolutionary generation of positional information de novo, enabling the translation of spatially homogeneous changes in signal levels into spatially heterogeneous (i.e. patterned) morphological change. Such cryptic patterns may be widespread in vertebrate skin, imposing a substantial bias on the types of morphological changes likely to occur from mutation and so be exposed to natural, sexual, and human selection.

## Materials and Methods

### Animals

The population used for mapping of the *Na* mutation, with 70 informative progeny, has been described [38]. Genotyping was performed with 11 additional microsatellite markers designed from the available chicken sequence assembly (Table S6). White Leghorn embryos were used as wild type controls for in situ hybridizations, quantitative RT-PCR, and skin explant cultures. Naked neck samples were obtained from England, Scotland, France, and Mexico. Additional DNA samples were obtained from the Transylvanian Naked Neck population provided by the Godollo Institute in Hungary to the AvianDiv collection. Wild type samples of various breeds were obtained from The Wernlas Collection, Shropshire, United Kingdom, and from the INRA collection of experimental lines. DNA was isolated from embryos or blood using proteinase K digestion, phenol/chloroform or high salt extraction, and ethanol precipitation. *Na/+* heterozygous embryos used to determine imbalanced allele expression were a cross between *Na/Na* and Silver Appenzeller. Oligonucleotides used for amplification across the indel polymorphism within the *BMP12* 3′UTR were: Forward: 5′-CGTGGTGTACAAACAGTACG-3′; Reverse: 5′-AAGCCCGGCCTTTTTATAGC-3′. PCR products were purified (QIAGEN) and directly sequenced.

### In Situ Hybridization

Embryos or skin cultures were fixed overnight in 4% paraformaldehyde in PBS at 4°C. Samples were dehydrated into methanol, bleached using H_2_O_2_, rehydrated, treated with 5 µg/ml proteinase K, post-fixed, and hybridized. Samples were washed to remove unbound probe and hybridization detected using an alkaline phosphatase conjugated sheep anti-digoxigenin (Roche) and a BCIP/NBT color reaction.

### Quantitative RT-PCR

Total RNA was isolated using TRI reagent and reverse transcribed using random primers and AMV reverse transcriptase (Roche) in a 20 µl total volume. Reactions were diluted 10-fold and 5 µl used as template for each qPCR. Double dye (5′FAM, 3′TAMRA) probes and primers were supplied by Eurogentec and Applied Biosystems. Probe sequences used were: *GAPD*: 5′-FAM-CATCGATCTGAACTACATGGTTTA-TAMRA-3′; *BMP12*: 5′-FAM-TCGGCACCGTCACCGGCTTC-TAMRA-3′; *SOSTDC1*: 5′-FAM-ACTTGAACGCGATTGTTAC-TAMRA-3′; *DHRS3*: 5′-FAM-AGGCGAGGAGCCAGGAAGATCATCC-TAMRA-3′; *RALDH2*/*ALDH1A2*: 5′-FAM-CAGATGCTGATTTGGATTATGCTGT-TAMRA-3′; and *RALDH3*/*ALDH1A3*: 5′-FAM-TGAGGAAGGAGACAAGCCTGATGTG-TAMRA-3′.

Twenty-microliter reactions were performed in triplicate, with at least four biological replicates used to determine each data point. Relative levels of *GAPD, SOSTDC1, RALDH2, RALDH3,* and *DHRS3* were determined from a dilution standard curve, while a plasmid standard curve was used to determine *BMP12* levels.

### Organotypic Skin Culture and Pattern Morphometrics

Dorsal skin from the entire crown-caudal length of E7.0 White Leghorn embryos was dissected and placed onto an MF-Millipore filter on a metal grid and submerged in DMEM containing 2% FBS in a centre well dish (Falcon) at 37°C, 5% CO_2_. Recombinant human BMP4 and mouse BMP12 (R&D Systems) were used. Dorsomorphin, Citral, and all-trans retinoic acid were supplied by Sigma-Aldrich and SB203580 by Merck. Feather placode densities, shapes, and areas were measured on *β*-*catenin* hybridized skin samples using ImagePro PLUS (Mediacybernetics). Circular placodes were defined as *β*-*catenin* positive foci with a circularity ratio (perimeter^2^/4πarea) of ≤1.2. Placode densities were determined only within tracts and did not include non-feathered areas. Mathematical modeling methods are described in Text S1.

### Exon Sequencing

All predicted exons in the ENSEMBL database lying between chromosome 3: 104754409–105526289 were amplified by PCR from genomic DNA of individuals in the French *Na/Na* experimental population and directly sequenced using the primers used for PCR amplification (oligonucleotide sequences available on request). Functional variants were defined as non-synonymous, frameshift, or nonsense SNPs within a predicted open reading frame, or as nucleotide substitutions within 10 bases of an intron/exon junction, based on comparison to the reference genome. Putative functional variants that were not in the dbSNP database were then sequenced from wild type individuals. No functional variants that were unique to *Na/Na* were identified.

### Expression Arrays

The microarray study used the Agilent Chicken expression arrays (design 015068: Agilent Technologies, Berks, UK) in a two dye reference experiment. Neck skin total RNA was labeled with Cy5 and body skin total RNA was labeled with Cy3 using the Ambion MessageAMP kit with aminoallyl labeled UTP (Applied Biosystems, UK) and the Cy3 and Cy 5 Dyes (GE Healthcare Life Sciences, Bucks, UK) according to manufacturers' protocols. Four independent E7.0 White leghorn body/neck RNA pairs were used for independent, unpooled hybridizations, which were carried out using the Agilent hybridization chambers and equipment. The slides were washed according to Agilent Technologies protocols and scanned in an Axon 4200AL scanner (Molecular Devices, UK) at 10 micron resolution. The scanned images were processed using the Feature Extraction software from Agilent Technologies.

## Supporting Information

Figure S1 Fine mapping of the *Na* mutation. (A) Schematic of the *Na* critical region on chromosome 3, between SEQ0465 and SEQ0467. The region with conserved synteny in the human genome also contains five annotated genes, making it unlikely that other genes are either unannotated or present in gaps in the chicken genome sequence. The first exon and the intron of *BMP12* were not present in the available genome sequence. We filled this region by amplification of gaps from BAC clones followed by sequencing. (B) Haplotypes of non-recombinant (NR) or recombinant (R) individuals. The *Na* haplotype is depicted in red, wild type haplotypes are in green. The recombinants localize the causative mutation between SEQ0465 and SEQ0467. ADL237 and MCW040 are the previous limits of the critical interval [41]. (−1) represents a null allele. SEQ0406 and SEQ0410 were not informative in our families.
(JPG)

Figure S2 Whole mount in situ hybridization detecting expression of *BMP12* in (A) E6.5 skin and (B) E8.0 skin and feather placodes.
(JPG)

Figure S3 Expression levels of *Na* critical interval genes in embryonic skin. Quantitative RT-PCR to detect relative gene expression levels in E7.5 wild type and *Na/Na* neck skin. The expression level of wild type is used to normalize for each gene. *p* values for pairwise comparisons between wild type and *Na/Na* expression levels are given for each gene. Oligonucleotides and probes used were supplied by Applied Biosystems. The sequences were: CB043-E4E5F 5′-CTGGAGATGATGAAGCGAGCAT-3′; CB043-E4E5R 5′-GCGCTCTATCGTGGGAAACA; CB043-E4E5M1 5′-FAM-TTCAGGTCCTCCGCTCCGT-NFQ-3′ HS1BP3-E2E3F 5′-CAAAGCACAAACCTGAGGATGTTG-3′; HS1BP3-E2E3R 5′-AGCTCCTCTATCTCGCTGTACTT-3′; HS1BP3-E2E3M2 5′-FAM-CTTGGACACCATAAACTG-NFQ-3′; TDC6-ANYF 5′-GAAGATACCAGCACAAAAATTAATACATTTTCTGA-3′; TDC6-ANYR 5′-CTCCTCTATGCCACTGTCCATTT-3′; TDC6-ANYM2 5′-FAM-CAGCACAAAATTGC-NFQ-3′; APOB-E23F 5′-GCTGTGAATGCTGATTCTGTTTTTGA-3′; APOB-E23R 5′-GCACAAGTGAATCCATTTCTACTAGAAGA-3′; APOB-E23M2 5′-FAM-CCTCTCCAGAACCTTTC-NFQ-3′.
(JPG)

Figure S4 Map of insertion breakpoints in Naked neck chromosome 3. (A) Sequences of breakpoints obtained from PCR products shown in Figure S5. Sequencing primers were: Left end primer LER2: 5′-TTAAGGAGGGGAAGTGCAGA-3′; Right end primer HR7_138: 5′-ATCACCAAAGGCTCTTTCCA-3′. (B) Sequence traces at left and right insertion breakpoints showing chromosome 1 and chromosome 3 sequences, boxed in red and blue, respectively, together with unaligned nucleotides at the junctions. A “CA” dinucleotide present in wild type chromosome 3 at the insertion is absent from the mutant locus (underlined in sequence trace).
(DOC)

Figure S5 Confirmation of the presence of a large chromosome 1–derived insertion in chromosome 3 of Naked neck genomes. (A) Map of chromosome 3 and chromosome 1 regions from wild type and *Na/Na* with primers used for PCR indicated. (B) Agarose gel showing the PCR amplification products obtained from 2 wild type and 2 *Na/Na* individuals using the primers diagrammed in (A). Oligo sequences: HR7_137: 5′-TGCCTACAATCCAGGAGAAG-3′; HR7_138: 5′-ATCACCAAAGGCTCTTTCCA-3′; HR7_139: 5′-CCATAGGCACATAGGCAGGT-3′; HR7_140: 5′-AACACCATTTCCCAAAGCAG-3′; LEFlankChr1F: 5′-GGTCAGCTGTCTGGGTACTGA-3′; LER3: 5′-GAGCCTGGACTACTCGCATC-3′; REF3: 5′-CTTGCTCAAGAGCCAGGAAG-3′; REFlankChr1R: 5′-CTAAGCCGGGACTCCTTCTT-3′.
(JPG)

Figure S6 Ex vivo recapitulation of the Naked neck phenotype upon application of recombinant BMP proteins. Embryonic skin explants were treated with 80 ng/ml recombinant BMP12 or BMP4. Treatment with either BMP family member abolished neck feathering and reduced feather row number on the body while allowing feather development on the head skin, as observed in the Naked neck phenotype.
(JPG)

Figure S7 Simulated patterning fields with different gradients of Inhibitor sensitivity display distinct behaviors when subjected to increasing Inhibitor concentrations. (A–F) Show the slope of the Inhibitor sensitivity gradient (α, ranging from 0.5 to 10) and the corresponding pattern behavior upon increasing ubiquitous Inhibitor concentration (*C_I_*). (A) A shallow gradient of Inhibitor sensitivity yields a receding boundary between head and neck as Inhibitor concentration is increased, a phenomenon not observed in BMP application experiments. (B–E) Sharpening of the gradient yields a stable boundary between head and neck with increasing Inhibitor concentration, consistent with experimental results. (F) A very sharp gradient, approximating a step change between body and neck Inhibitor sensitivities, produces a distinct aligned row of Activator foci at the boundary between neck and body at all concentrations of Inhibitor. Such an alignment of foci along the neck/body boundary is not observed in untreated chicken skin.
(JPG)

Figure S8The range of patterns produced by our reaction-diffusion model, as predicted by the parameter sensitivity analysis. (A) The pattern produced with our default parameter set (Table S3) using 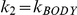 across a field of dimensions 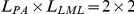. (B) A similar pattern is produced despite a 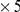 perturbation of 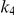. (C,D) Examples of (C), decreased placode density following a 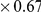 perturbation of 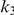 and (D), increased placode density following a 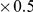 perturbation of 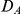. (E,F) Examples of fusions/stripes for an (E), 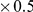 perturbation of 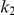 or (F), 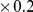 perturbation of 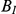. (G,H) Examples showing (G), ubiquitously high Activator for a 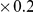 perturbation of 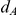 or (H), ubiquitously low Activator for a 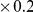 perturbation of 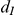.(JPG)

Figure S9 Lateral view of RA pathway gene expression in embryonic skin. (A–C) *RALDH2*, *RALDH3*, and *DHRS3* expression in E7.5 embryos. In addition to the lateral aspect of the neck, prominent staining is seen on the limbs, particularly on the hindlimb at the margin of the presumptive femoral feather tract. (D) Detection of *β-catenin* expression at E8.5 illustrates the extent of the femoral tract (arrow), with the RA-active region on the hindlimb lying distal to the site of feather patterning.
(JPG)

Figure S10 Selective expression of retinoic acid pathway genes on the neck across avian species. Whole mount in situ hybridization detecting expression of (A–D) the RA target gene *DHRS3* and the RA synthesizing enzymes (E–H) *RALDH2* and (I–L) *RALDH3* during feather patterning in duck, quail, guinea fowl, and turkey embryos. RA responses are detected on the neck in all species. In duck the boundary between RA-high and RA-low skin lies more anteriorly than in other species, and *RALDH3* expression shows little difference between neck and body, while *RALDH2* displays intense signal on the neck. (M–P) Detection of *β-catenin* expression, indicating the stage of feather patterning, in each species. Scale bars indicate 2 mm.
(JPG)

Figure S11 Equalization of neck and body feather density as a result of post-patterning skin growth. In E9.5 embryos, the density of placodes on the neck is 33% lower than that on the body, similar to observations in cultured skin (Figure 3). In adult neck and body skin the feather density is approximately equal. This equalization of follicle density on neck and body is a result of differential growth of these two regions following the laying out of the embryonic placode pattern, which causes a greater “stretching” of the pattern on the body than the neck. Embryonic placode density was determined by detection of placodes using *β-catenin* in situ hybridization on E9.5 embryos, followed by dissection of skin, flattening onto a glass slide, photography, and measurement of placode density per square millimeter. Determination of feather density in mature skin was done using 6-mo-old female hens. Feathers were plucked from the spinal tract (neck and body) to reveal the follicles. Skin was peeled off the body and flattened, then photographed, and feather follicle density per square centimeter determined. Placode or follicle density was measured in the spinal feather tract only. Three animals were used for density measurement at each age. Error bars indicate S.E.M.
(JPG)

Table S1 Identification of an identical by descent (IBD) region in Naked neck individuals. Genotyping results for *Na/+*, *Na/Na*, and wild type individuals for markers lying within the mapped *Na* critical interval, which is defined by markers SEQ0465 and SEQ0467. Known SNPs are labeled according to SNP ID, and the chromosome 3 coordinate of the SNP, or the initial nucleotide coordinate for sequence length polymorphisms, is given below. Previously undescribed SNPs are labeled in italics according to our marker names. Each marker was amplified by PCR and the subsequent genotyping assay is indicated: SEQ, direct sequencing of PCR product; CAPS, restriction enzyme cleavage of PCR product with the relevant enzyme following in parentheses; GE, gel electrophoresis for simple length polymorphisms. Sequences of oligonucleotides used to amplify each marker are given below the assay type. Individuals not typed or reaction fails are indicated by “-”. The IBD region defined is 201,657 bp on chromosome 3 of the reference genome, spanning nucleotide coordinates 104925030 to 105126687.
(XLS)

Table S2 Genotyping results for chromosome 1 insertion into chromosome 3. Results of a triplex PCR including primers across the insertion breakpoint on chromosome 3, and from the chromosome 1 insertion to flanking chromosome 3 sequence, are shown in the upper part of the table. The insertion is not detected in any wild type individuals, and amplification across the insertion breakpoint occurs in *Na/Na* individuals. Below are the results of two independent PCR assays to detect insertion right and left ends. These amplified from all Naked neck individuals tested and not from any wild type individuals. Oligonucleotide information and predicted PCR product sizes are given for each assay.
(XLS)

Table S3Model parameters, their phenomenological descriptions, and their (nondimensional) default values for the numerical simulations of Figure 3 and Figure 4 and the sensitivity analysis. For the simulations presented in Figure 3, parameter 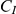 is varied between 0.0 (default) and 1.0 to represent increasing doses of an exogenous Inhibitor. For the simulations in Figure 4, 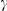 is decreased from 1.0 (default) to 0.05 to represent a varying degree of suppression of Inhibitor activity.(DOC)

Table S4Results from a parameter perturbation analysis of the model. Simulations were performed as described in the methods with each parameter individually perturbed from its default value listed in Table S3 (here we set 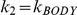 and 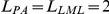) by the factor tabulated in the first row. The density/form of the placode pattern was compared at the end of the simulation against that produced by the default parameter using the following classifications: (-) “normal patterning”—placode density deviates <15% from default parameter set; (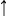) placode density increases >15%; (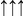) placode density increases >50%; (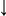) placode density decreases >15%; (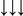) placode density decreases >50%; (F) placode fusions/stripes; (0) ubiquitously low activator—no pattern; (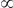) ubiquitously high activator—no pattern. Representative examples of these various pattern types are provided in Figure S8.(DOC)

Table S5 Probes and corresponding gene names showing the greatest fold expression differences between E7.0 neck and body skin on Agilent expression array. Sequences were mapped onto the reference genome by BLAT search and gene name indicates the overlapping or closest transcriptional unit in the ENSEMBL browser.
(XLS)

Table S6 Microsatellite markers developed for mapping of the *Na* locus. *May 2006 chicken (*Gallus gallus*) v2.1 draft assembly, UCSC (http://genome.ucsc.edu/cgi-bin/hgGateway).
(DOC)

Text S1 Mathematical modelling.
(DOC)

## Abbreviations

BMP: bone morphogenetic protein
GDF: growth and differentiation factor
kb: kilobase pairs
Na: Naked neck
RA: retinoic acid
RALDH: retinaldehyde dehydrogenase
SNP: single nucleotide polymorphism
UTR: untranslated region
