# Supplementary figures and images for: Cryptic Patterning of Avian Skin Confers a Developmental Facility for Loss of Neck Feathering
Source: PLoS Biol. 2011 Mar 15;9(3):e1001028. doi: 10.1371/journal.pbio.1001028 (PMC3057954; doi:10.1371/journal.pbio.1001028)

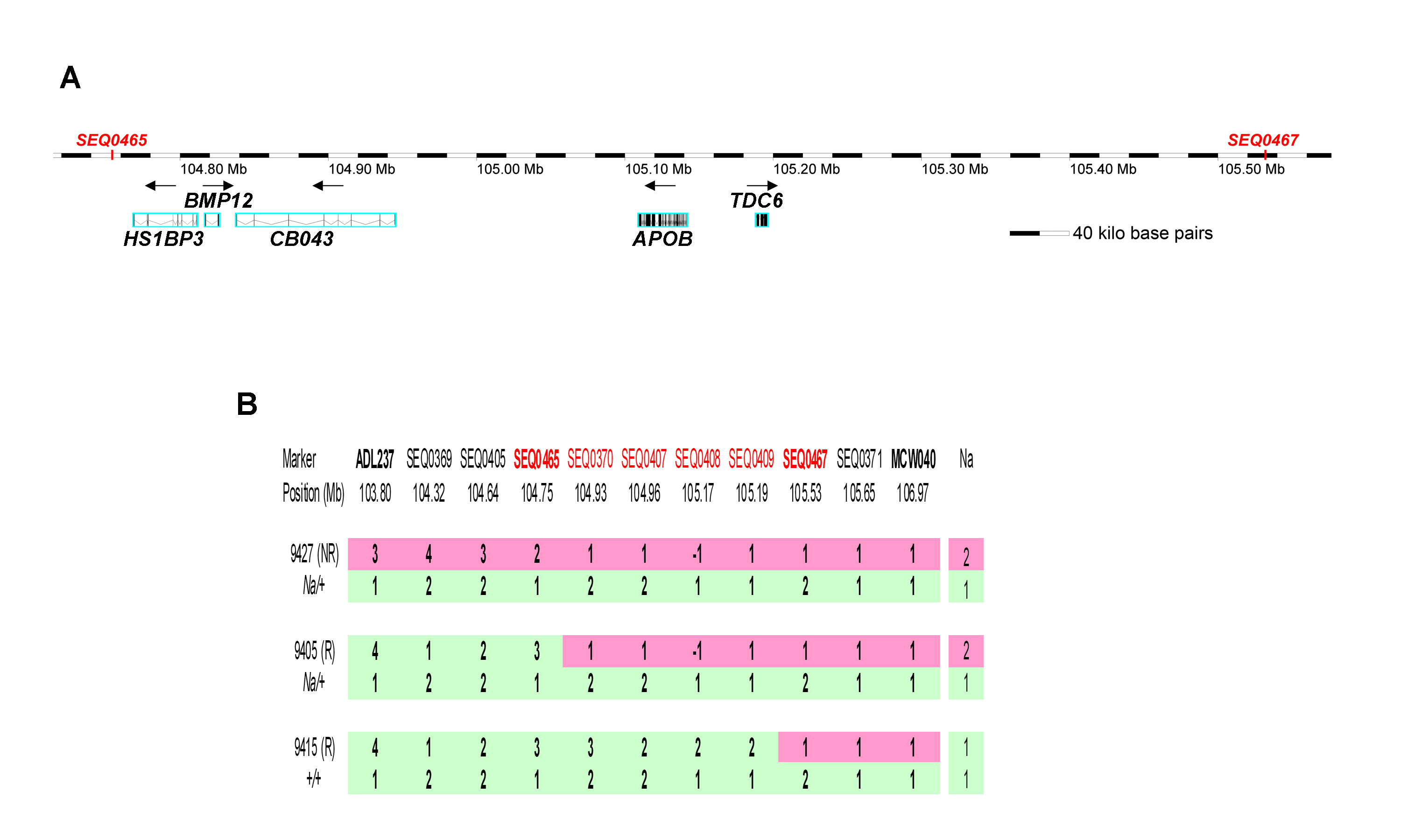

Supplement: Figure S1 — Fine mapping of the Na mutation. (A) Schematic of the Na critical region on chromosome 3, between SEQ0465 and SEQ0467. The region with conserved synteny in the human genome also contains five annotated genes, making it unlikely that other genes are either unannotated or present in gaps in the chicken genome sequence. The first exon and the intron of BMP12 were not present in the available genome sequence. We filled this region by amplification of gaps from BAC clones followed by sequencing. (B) Haplotypes of non-recombinant (NR) or recombinant (R) individuals. The Na haplotype is depicted in red, wild type haplotypes are in green. The recombinants localize the causative mutation between SEQ0465 and SEQ0467. ADL237 and MCW040 are the previous limits of the critical interval [41]. (−1) represents a null allele. SEQ0406 and SEQ0410 were not informative in our families. (JPG) [file pbio.1001028.s001.jpg]

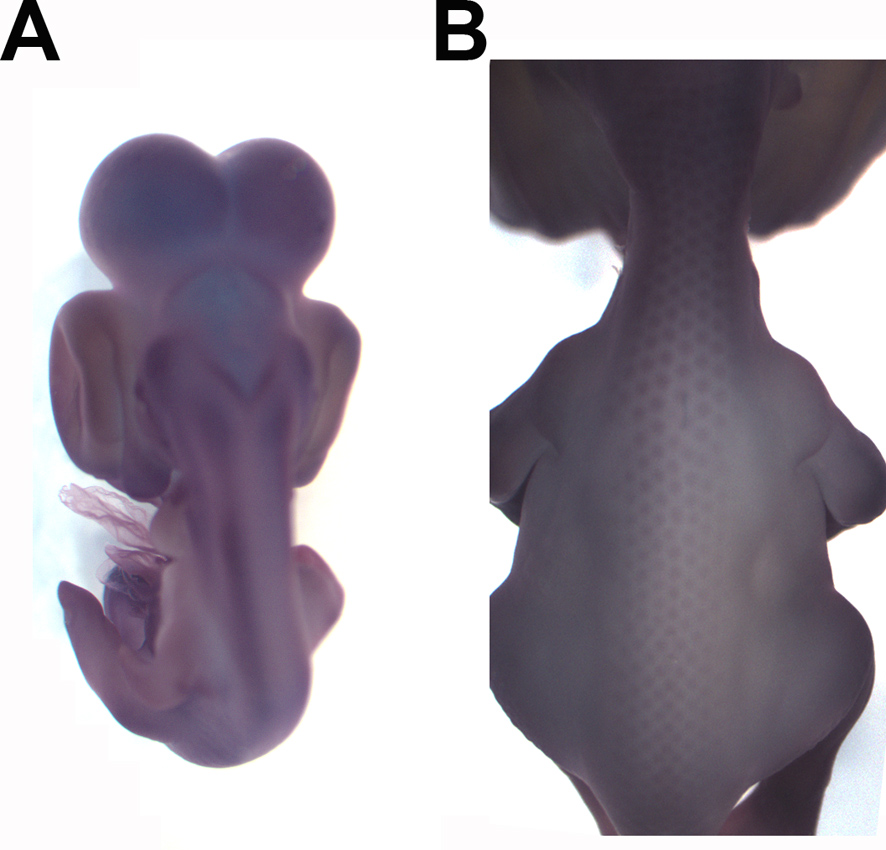

Supplement: Figure S2 — Whole mount in situ hybridization detecting expression of BMP12 in (A) E6.5 skin and (B) E8.0 skin and feather placodes. (JPG) [file pbio.1001028.s002.jpg]

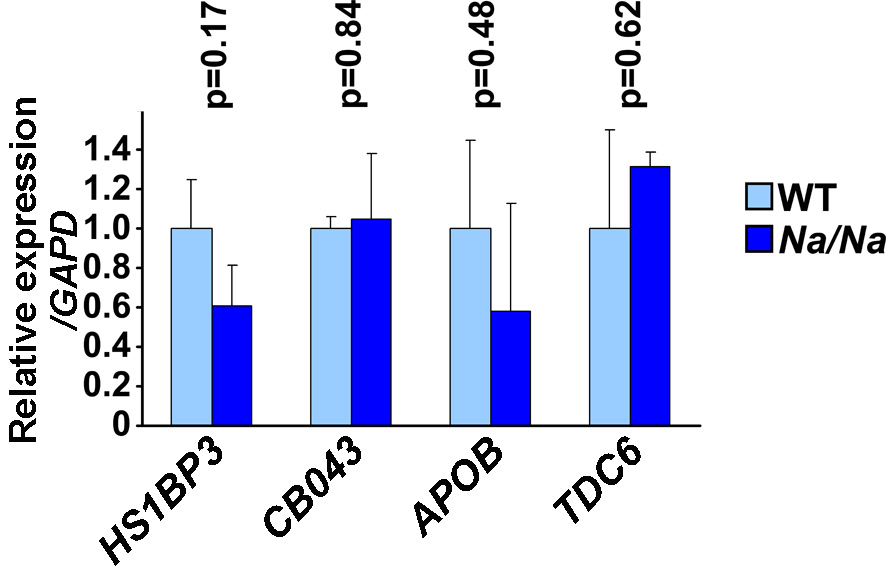

Supplement: Figure S3 — Expression levels of Na critical interval genes in embryonic skin. Quantitative RT-PCR to detect relative gene expression levels in E7.5 wild type and Na/Na neck skin. The expression level of wild type is used to normalize for each gene. p values for pairwise comparisons between wild type and Na/Na expression levels are given for each gene. Oligonucleotides and probes used were supplied by Applied Biosystems. The sequences were: CB043-E4E5F 5′-CTGGAGATGATGAAGCGAGCAT-3′; CB043-E4E5R 5′-GCGCTCTATCGTGGGAAACA; CB043-E4E5M1 5′-FAM-TTCAGGTCCTCCGCTCCGT-NFQ-3′ HS1BP3-E2E3F 5′-CAAAGCACAAACCTGAGGATGTTG-3′; HS1BP3-E2E3R 5′-AGCTCCTCTATCTCGCTGTACTT-3′; HS1BP3-E2E3M2 5′-FAM-CTTGGACACCATAAACTG-NFQ-3′; TDC6-ANYF 5′-GAAGATACCAGCACAAAAATTAATACATTTTCTGA-3′; TDC6-ANYR 5′-CTCCTCTATGCCACTGTCCATTT-3′; TDC6-ANYM2 5′-FAM-CAGCACAAAATTGC-NFQ-3′; APOB-E23F 5′-GCTGTGAATGCTGATTCTGTTTTTGA-3′; APOB-E23R 5′-GCACAAGTGAATCCATTTCTACTAGAAGA-3′; APOB-E23M2 5′-FAM-CCTCTCCAGAACCTTTC-NFQ-3′. (JPG) [file pbio.1001028.s003.jpg]

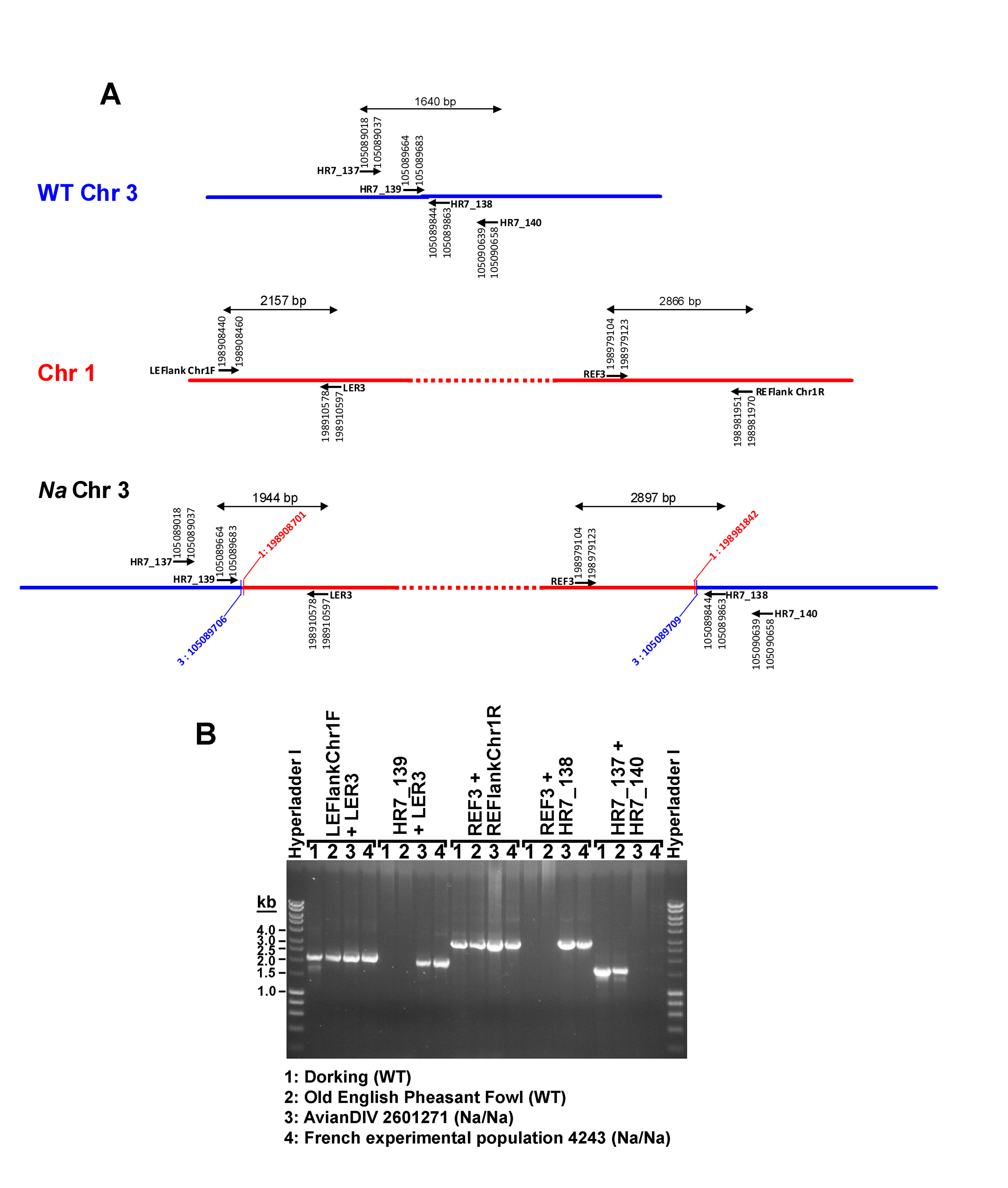

Supplement: Figure S5 — Confirmation of the presence of a large chromosome 1–derived insertion in chromosome 3 of Naked neck genomes. (A) Map of chromosome 3 and chromosome 1 regions from wild type and Na/Na with primers used for PCR indicated. (B) Agarose gel showing the PCR amplification products obtained from 2 wild type and 2 Na/Na individuals using the primers diagrammed in (A). Oligo sequences: HR7_137: 5′-TGCCTACAATCCAGGAGAAG-3′; HR7_138: 5′-ATCACCAAAGGCTCTTTCCA-3′; HR7_139: 5′-CCATAGGCACATAGGCAGGT-3′; HR7_140: 5′-AACACCATTTCCCAAAGCAG-3′; LEFlankChr1F: 5′-GGTCAGCTGTCTGGGTACTGA-3′; LER3: 5′-GAGCCTGGACTACTCGCATC-3′; REF3: 5′-CTTGCTCAAGAGCCAGGAAG-3′; REFlankChr1R: 5′-CTAAGCCGGGACTCCTTCTT-3′. (JPG) [file pbio.1001028.s005.jpg]

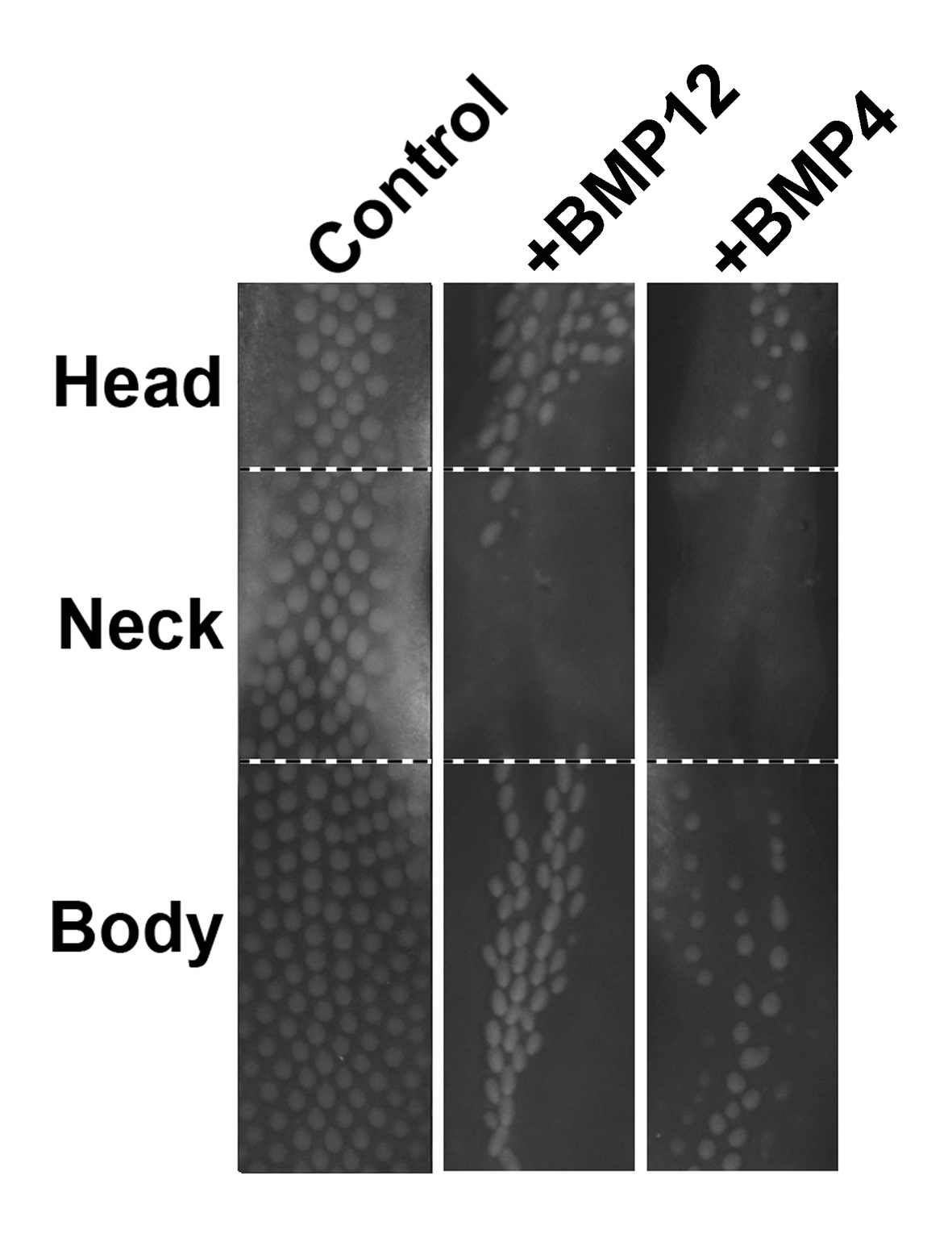

Supplement: Figure S6 — Ex vivo recapitulation of the Naked neck phenotype upon application of recombinant BMP proteins. Embryonic skin explants were treated with 80 ng/ml recombinant BMP12 or BMP4. Treatment with either BMP family member abolished neck feathering and reduced feather row number on the body while allowing feather development on the head skin, as observed in the Naked neck phenotype. (JPG) [file pbio.1001028.s006.jpg]

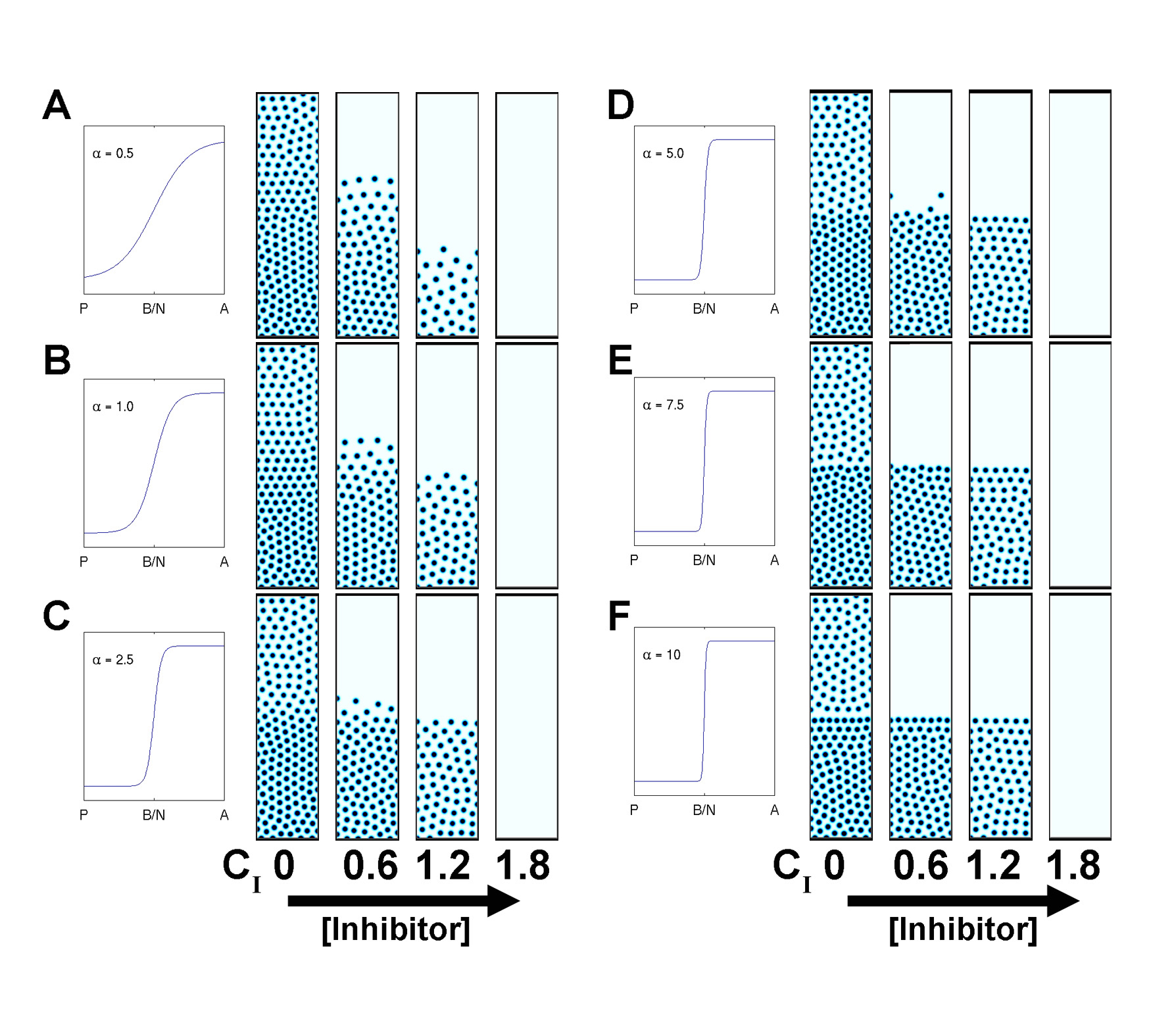

Supplement: Figure S7 — Simulated patterning fields with different gradients of Inhibitor sensitivity display distinct behaviors when subjected to increasing Inhibitor concentrations. (A–F) Show the slope of the Inhibitor sensitivity gradient (α, ranging from 0.5 to 10) and the corresponding pattern behavior upon increasing ubiquitous Inhibitor concentration (CI). (A) A shallow gradient of Inhibitor sensitivity yields a receding boundary between head and neck as Inhibitor concentration is increased, a phenomenon not observed in BMP application experiments. (B–E) Sharpening of the gradient yields a stable boundary between head and neck with increasing Inhibitor concentration, consistent with experimental results. (F) A very sharp gradient, approximating a step change between body and neck Inhibitor sensitivities, produces a distinct aligned row of Activator foci at the boundary between neck and body at all concentrations of Inhibitor. Such an alignment of foci along the neck/body boundary is not observed in untreated chicken skin. (JPG) [file pbio.1001028.s007.jpg]

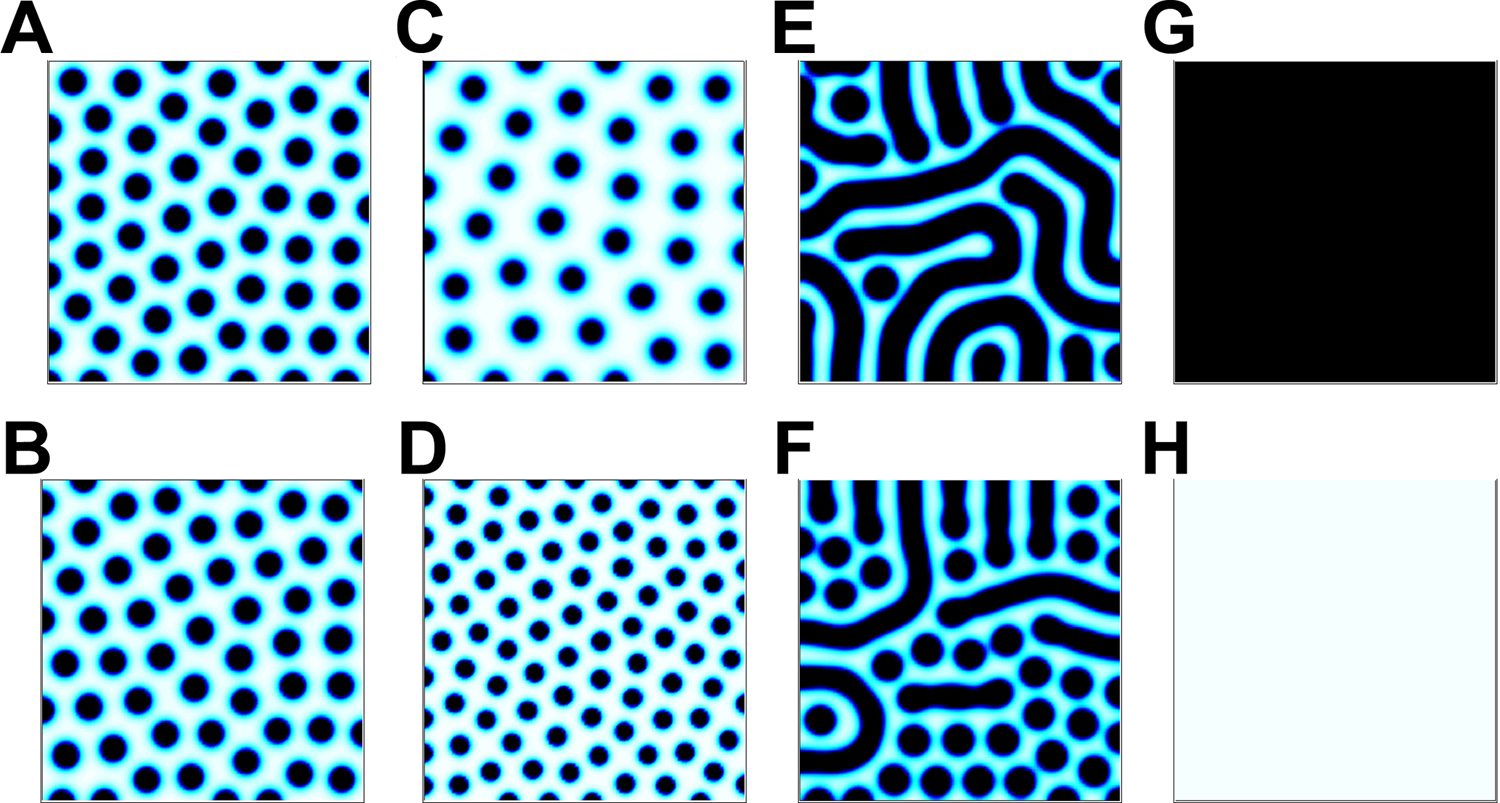

Supplement: Figure S8 — The range of patterns produced by our reaction-diffusion model, as predicted by the parameter sensitivity analysis. (A) The pattern produced with our default parameter set (Table S3) using across a field of dimensions . (B) A similar pattern is produced despite a perturbation of . (C,D) Examples of (C), decreased placode density following a perturbation of and (D), increased placode density following a perturbation of . (E,F) Examples of fusions/stripes for an (E), perturbation of or (F), perturbation of . (G,H) Examples showing (G), ubiquitously high Activator for a perturbation of or (H), ubiquitously low Activator for a perturbation of . (JPG) [file pbio.1001028.s008.jpg]

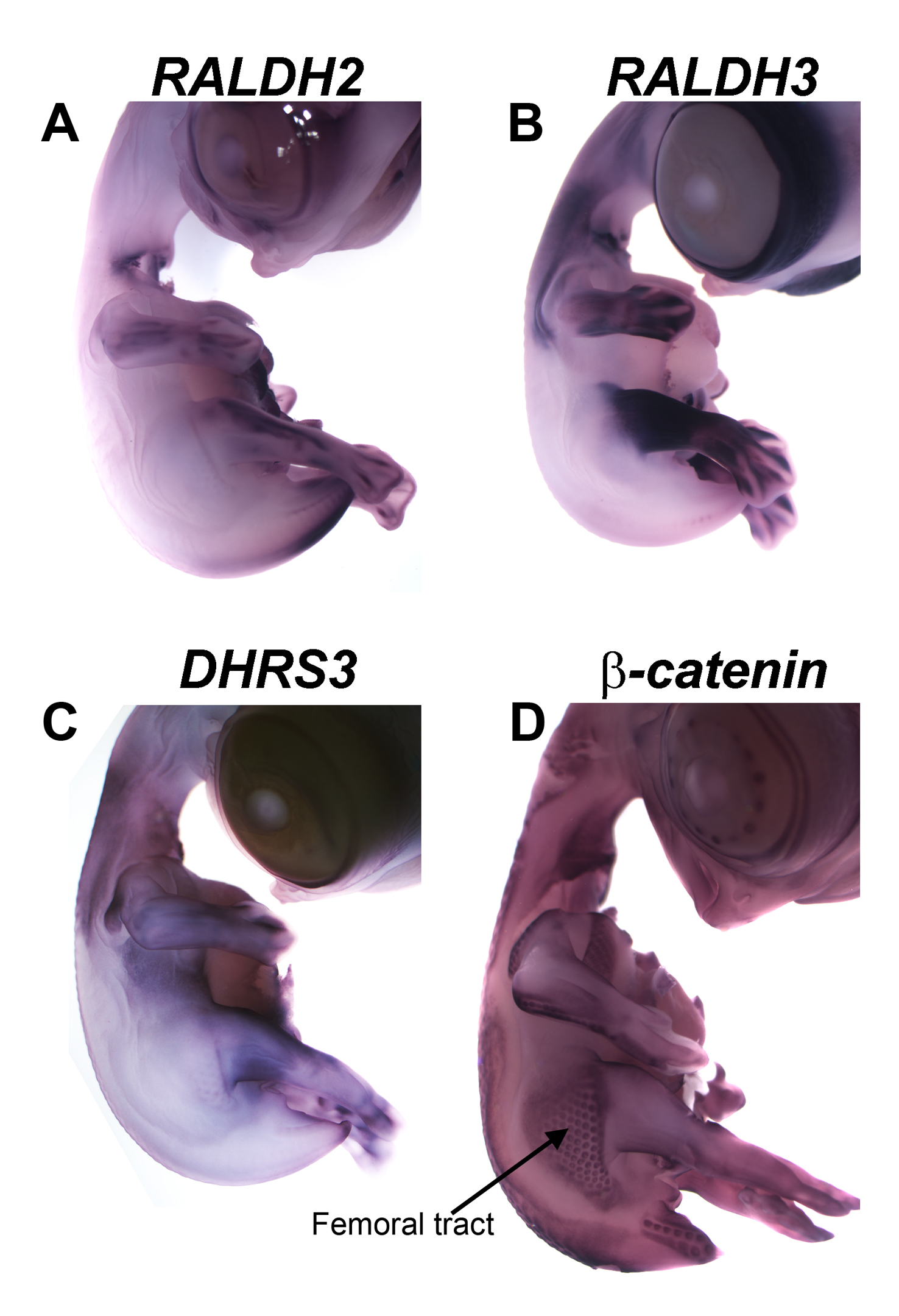

Supplement: Figure S9 — Lateral view of RA pathway gene expression in embryonic skin. (A–C) RALDH2, RALDH3, and DHRS3 expression in E7.5 embryos. In addition to the lateral aspect of the neck, prominent staining is seen on the limbs, particularly on the hindlimb at the margin of the presumptive femoral feather tract. (D) Detection of β-catenin expression at E8.5 illustrates the extent of the femoral tract (arrow), with the RA-active region on the hindlimb lying distal to the site of feather patterning. (JPG) [file pbio.1001028.s009.jpg]

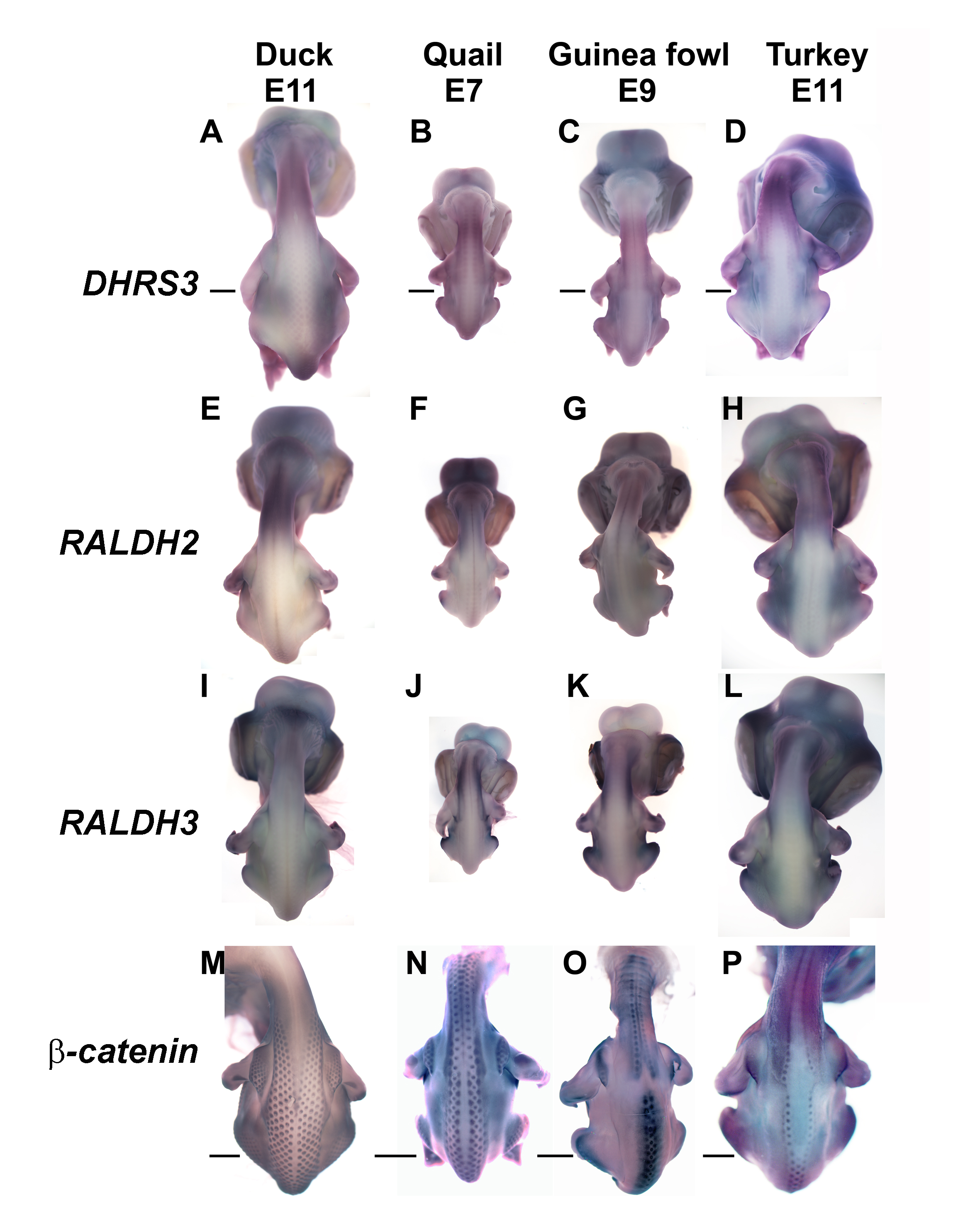

Supplement: Figure S10 — Selective expression of retinoic acid pathway genes on the neck across avian species. Whole mount in situ hybridization detecting expression of (A–D) the RA target gene DHRS3 and the RA synthesizing enzymes (E–H) RALDH2 and (I–L) RALDH3 during feather patterning in duck, quail, guinea fowl, and turkey embryos. RA responses are detected on the neck in all species. In duck the boundary between RA-high and RA-low skin lies more anteriorly than in other species, and RALDH3 expression shows little difference between neck and body, while RALDH2 displays intense signal on the neck. (M–P) Detection of β-catenin expression, indicating the stage of feather patterning, in each species. Scale bars indicate 2 mm. (JPG) [file pbio.1001028.s010.jpg]

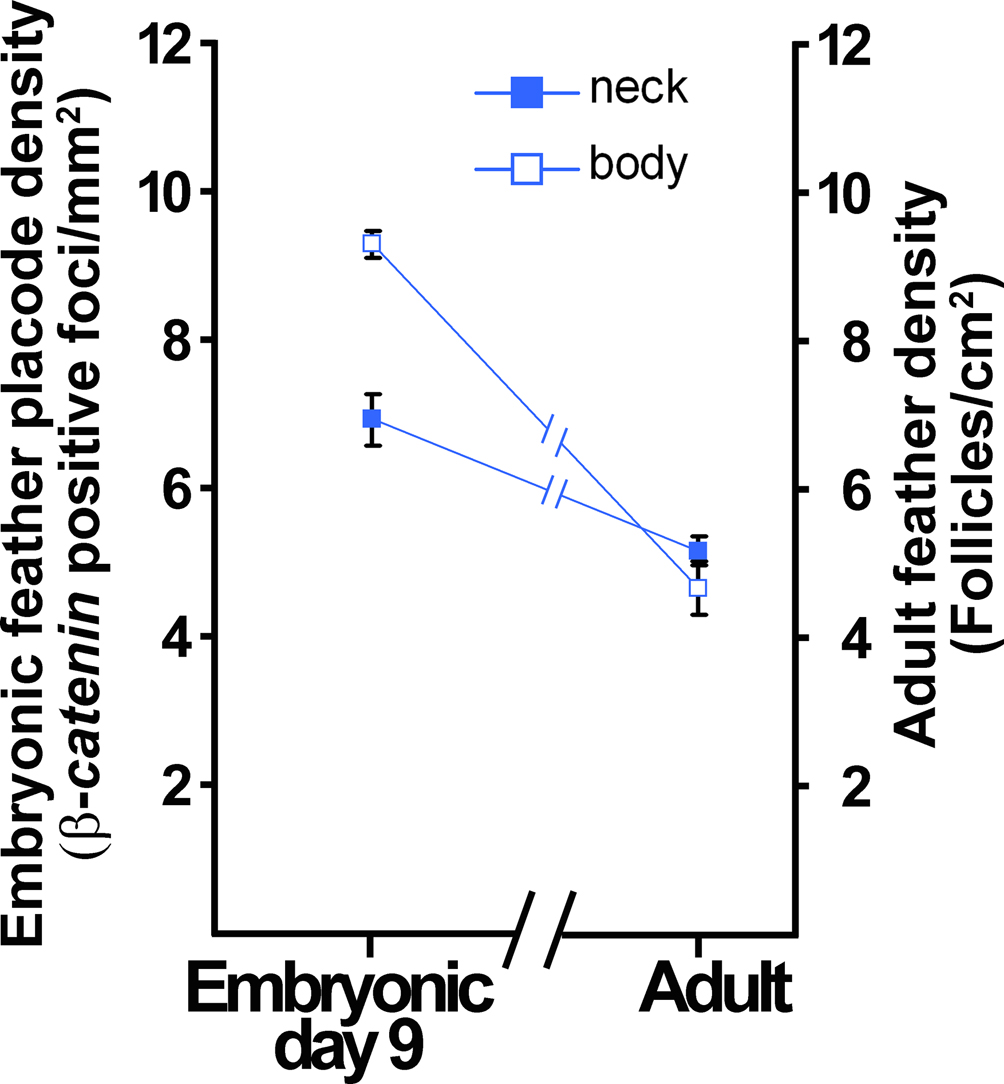

Supplement: Figure S11 — Equalization of neck and body feather density as a result of post-patterning skin growth. In E9.5 embryos, the density of placodes on the neck is 33% lower than that on the body, similar to observations in cultured skin (Figure 3). In adult neck and body skin the feather density is approximately equal. This equalization of follicle density on neck and body is a result of differential growth of these two regions following the laying out of the embryonic placode pattern, which causes a greater “stretching” of the pattern on the body than the neck. Embryonic placode density was determined by detection of placodes using β-catenin in situ hybridization on E9.5 embryos, followed by dissection of skin, flattening onto a glass slide, photography, and measurement of placode density per square millimeter. Determination of feather density in mature skin was done using 6-mo-old female hens. Feathers were plucked from the spinal tract (neck and body) to reveal the follicles. Skin was peeled off the body and flattened, then photographed, and feather follicle density per square centimeter determined. Placode or follicle density was measured in the spinal feather tract only. Three animals were used for density measurement at each age. Error bars indicate S.E.M. (JPG) [file pbio.1001028.s011.jpg]
