# Supplementary material for: Cryptic Patterning of Avian Skin Confers a Developmental Facility for Loss of Neck Feathering
Source: PLoS Biol. 2011 Mar 15;9(3):e1001028. doi: 10.1371/journal.pbio.1001028 (PMC3057954; doi:10.1371/journal.pbio.1001028)

**A**

**Chromosome 3 sequence**

**Chromosome 1 sequence**

Nucleotides that do not align to the reference genome are italicised

**Left junction**

**TCCATAGGCACATAGGCAGGTGGGAACCCAAATAAAGGGCTAAG***GTAAAG***TGCCCCTTCACTTTACCAGCGAATTCAGCCAAAATGAAGTTGGAAACCCTTTGCCTCTGCTCTTGTAAAGCAAAGCTTCCCAAATTCATGGTAAATAAATTTCTCCTGATGTCCACTCTGATCCTCCCCTGGCACAACTTTGTGCCGTTCCTGCGTGTCCTGTCATCGGTTCCAGGAGAGGAGAAGGCCCCAATCC**

**Right junction**

**CAATGAAAAATTTACCCTTTAACTGTGTATATTTTTATGGAGAAAGGTTATCTATCCATCCATCATCACATACAAGTACAGGAAAGAGCCTGTGAGTATTTAGATATTATATCTTGCATGTGTAGTTAAAGAGAAATAATAGTAGCACAGAGACGGCTGTATCTGCTCCCTCTCTCCTCTCCGTTTAGGTGTCCACTGCAAAAACTTCATACATAAATTGAAGCTGCTCATCTTATAGATTTAATCATCTATATTAGGATGAAATGAATGGCAGTAACTGTATGGACTCCCCCATCGGCAACGAACAAAACAGGGAATCAAGAGAGCCATCCCCAGAGCAGGTGTTTCTCTTGCACAAAGGATGCTGTGGCCACCCAACATCTCCAGTCCATGGGTAGTAGCTGCCTTCTCAAGGCCATTGAAACCCACTTTTCTGGGTCTGGAGCACGGGGACAGGTCTC***AATGG***GAAGGAACATAATCTCTAGGCTCTCCCAGGAGATTCTCAGCTCCAGTGTGCAAGTGTGGCACCCATCACAAGCCAGTATTGAGGGCAGCTTTGGACTTGGGTGTGACCAT**

**B**


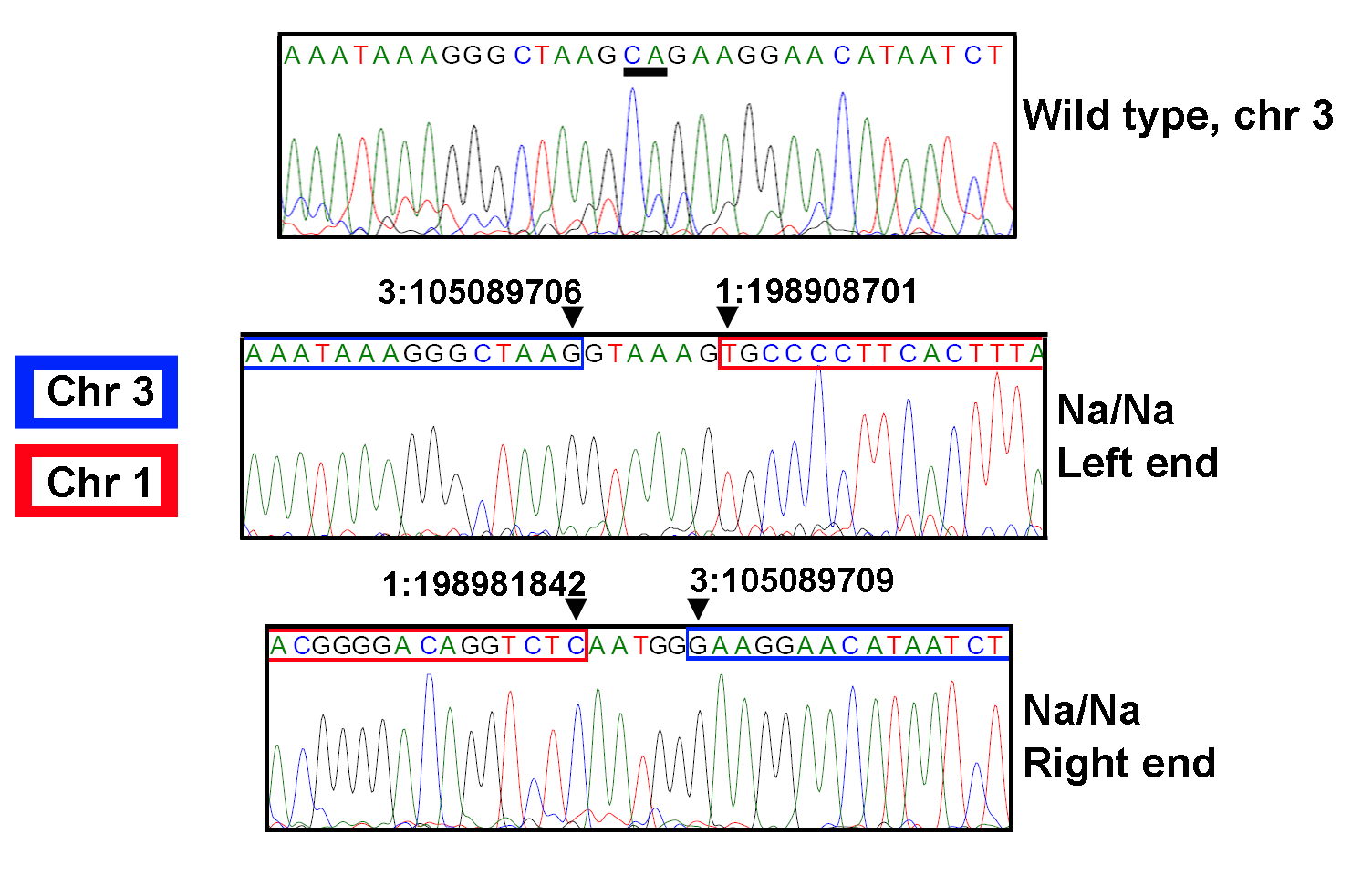

Supplement: Figure S4 — Map of insertion breakpoints in Naked neck chromosome 3. (A) Sequences of breakpoints obtained from PCR products shown in Figure S5. Sequencing primers were: Left end primer LER2: 5′-TTAAGGAGGGGAAGTGCAGA-3′; Right end primer HR7_138: 5′-ATCACCAAAGGCTCTTTCCA-3′. (B) Sequence traces at left and right insertion breakpoints showing chromosome 1 and chromosome 3 sequences, boxed in red and blue, respectively, together with unaligned nucleotides at the junctions. A “CA” dinucleotide present in wild type chromosome 3 at the insertion is absent from the mutant locus (underlined in sequence trace). (DOC) [file pbio.1001028.s004.doc]
