# Supplementary material for: Cryptic Patterning of Avian Skin Confers a Developmental Facility for Loss of Neck Feathering
Source: PLoS Biol. 2011 Mar 15;9(3):e1001028. doi: 10.1371/journal.pbio.1001028 (PMC3057954; doi:10.1371/journal.pbio.1001028)
Supplement: Table S3 — Model parameters, their phenomenological descriptions, and their (nondimensional) default values for the numerical simulations of Figure 3 and Figure 4 and the sensitivity analysis. For the simulations presented in Figure 3, parameter is varied between 0.0 (default) and 1.0 to represent increasing doses of an exogenous Inhibitor. For the simulations in Figure 4, is decreased from 1.0 (default) to 0.05 to represent a varying degree of suppression of Inhibitor activity. (DOC) [file pbio.1001028.s014.doc]

| **Notation** | **Interpretation** | **Default value** |
| --- | --- | --- |
| $p_{A}$ | Production/activity of Activator (A) | 10.0 |
| $p_{I}$ | Production/activity of Inhibitor (I) | 1.0 |
| $D_{A}$ | Diffusion rate/signaling range of Activator | 0.00025 |
| $D_{I}$ | Diffusion rate/signaling range of Inhibitor | 0.0125 |
| $d_{A}$ | Decay rate of Activator | 1.0 |
| $d_{I}$ | Decay rate of Inhibitor | 1.0 |
| $B_{I}$ | Background Inhibitor production | 4.3 |
| $C_{I}$ | Constitutive Inhibitor application parameter | 0.0-1.0 |
| $k_{1}$ | Parameter for Activator autocatalysis | 0.1 |
| $k_{2}$ | Sensitivity to inhibition by Inhibitor | 2.5-3.0 |
| $k_{BODY}$ | Sensitivity to inhibition by Inhibitor in body | 2.5 |
| $k_{NECK}$ | Sensitivity to inhibition by Inhibitor in neck | 3.0 |
| $k_{3}$ | Parameter for inhibition of Inhibition | 1.0 |
| $k_{4}$ | Parameter for upregulation of Inhibitor | 0.01 |
| $\gamma$ | Suppression of BMP/Inhibitor activity parameter | 1.0 - 0.05 |
| $\alpha$ | Steepness of sensitivity gradient | 7.5 |
| $L_{PA}$ | Length of posterior-anterior axis | 8.0 |
| $L_{LML}$ | Length of lateral-medial-lateral axis | 2.0 |
