# Supplementary material for: Cryptic Patterning of Avian Skin Confers a Developmental Facility for Loss of Neck Feathering
Source: PLoS Biol. 2011 Mar 15;9(3):e1001028. doi: 10.1371/journal.pbio.1001028 (PMC3057954; doi:10.1371/journal.pbio.1001028)
Supplement: Table S4 — Results from a parameter perturbation analysis of the model. Simulations were performed as described in the methods with each parameter individually perturbed from its default value listed in Table S3 (here we set and ) by the factor tabulated in the first row. The density/form of the placode pattern was compared at the end of the simulation against that produced by the default parameter using the following classifications: (-) “normal patterning”—placode density deviates <15% from default parameter set; () placode density increases >15%; () placode density increases >50%; () placode density decreases >15%; () placode density decreases >50%; (F) placode fusions/stripes; (0) ubiquitously low activator—no pattern; () ubiquitously high activator—no pattern. Representative examples of these various pattern types are provided in Figure S8. (DOC) [file pbio.1001028.s015.doc]

| $\times0.2$ | $\times0.5$ | $\times2/3$ | $\times0.8$ | $\times0.9$ | $\times1$ | $\times1.1$ | $\times1.25$ | $\times1.5$ | $\times2$ | $\times5$ |
| --- | --- | --- | --- | --- | --- | --- | --- | --- | --- | --- |
| 0 | 0 | 0 | 0 | - | $p_{A}$ | - | - | - | F | F |
| F | - | - | - | - | $p_{I}$ | - | - | - |  | 0 |
|  |  |  | - | - | $D_{A}$ | - | - |  |  | 0 |
| 0 |  | - | - | - | $D_{I}$ | - | - |  |  |  |
| $\infty$ | $\infty$ | F | - | - | $d_{A}$ | - | 0 | 0 | 0 | 0 |
| 0 | 0 | 0 | 0 | - | $d_{I}$ | - | - | F | $\infty$ | $\infty$ |
| F | - | - | - | - | $B_{I}$ | - |  | 0 | 0 | 0 |
| - | - | - | - | - | $k_{1}$ | - | - |  |  | 0 |
| $\infty$ | F | F | - | - | $k_{2}$ | - |  | 0 | 0 | 0 |
| 0 | 0 |  |  | - | $k_{3}$ | - | - | - | - | F |
| - | - | - | - | - | $k_{4}$ | - | - | - | - | - |
