# Supplementary material for: Cryptic Patterning of Avian Skin Confers a Developmental Facility for Loss of Neck Feathering
Source: PLoS Biol. 2011 Mar 15;9(3):e1001028. doi: 10.1371/journal.pbio.1001028 (PMC3057954; doi:10.1371/journal.pbio.1001028)
Supplement: Table S6 — Microsatellite markers developed for mapping of the Na locus. *May 2006 chicken (Gallus gallus) v2.1 draft assembly, UCSC (http://genome.ucsc.edu/cgi-bin/hgGateway). (DOC) [file pbio.1001028.s017.doc]

| Marker | Primer1 | Primer2 | Position* |
| --- | --- | --- | --- |
| SEQ0369 | GCATTTCCTTTGAATTTTACTTG | TGGTGGGCTGCTACTGTC | chr3:104321991+104322187 |
| SEQ0370 | TGACCCTCAACAACCAACAG | ATATAGCATTTTTGGGGAAGC | chr3:104927045+104927268 |
| SEQ0371 | ACTTGCCACGGGTTATTTTC | AGGGCAGCTACTTGCTTTAG | chr3:105649621+105649787 |
| SEQ0405 | TGTTCCTGCTTTCCTGAGC | CATCAAAATGCCTGGCTACC | chr3:104643891+104644138 |
| SEQ0406 | GCAATTTTTGCAGCTCTTCC | AGGAGCAGAGAGGGTTTCC | chr3:104826795-104826930 |
| SEQ0407 | GACGTGTTTGTCCCTTTGTG | TGGGATTTCAAGACCAGGAG | chr3:104963604+104963732 |
| SEQ0408 | TTTCCCAAGCTACAGCATAC | TCATACTCAGTGGGACCAG | chr3:105165381+105165575 |
| SEQ0409 | CAATTTCTGACTGTTCTAGCC | TCACTCCTGAAGGCACAGC | chr3:105184984+105185117 |
| SEQ0410 | TCTACACTTTGACAATACGTC | ACTGGCATCGTACCTGTGC | chr3:105476498+105476775 |
| SEQ0465 | TTGTCAGATTGGCTTTGGAG | TAGGCAGCTGATGGGTTTTC | chr3:104754409+104754650 |
| SEQ0467 | TTCTCCCATCATTTCTTTTTCC | AGCTCAACAGCAGGGAAGAC | chr3:105526289+105526482 |
