# Supplementary material for: Cryptic Patterning of Avian Skin Confers a Developmental Facility for Loss of Neck Feathering
Source: PLoS Biol. 2011 Mar 15;9(3):e1001028. doi: 10.1371/journal.pbio.1001028 (PMC3057954; doi:10.1371/journal.pbio.1001028)
Supplement: Text S1 — Mathematical modelling. (DOC) [file pbio.1001028.s018.doc]

**Mathematical modelling**

To test the hypothesis that differential sensitivity to Inhibitor in a reaction-diffusion scheme is responsible for the distinct responses between neck and body to varying concentrations of applied BMP12, we developed a mathematical model and performed *in silico* modeling of feather placode formation. The model belongs to the general Activator-Inhibitor/Turing class of reaction-diffusion equations [see ref.s 1,2] and phenomenologically describes the core set of key interactions schematized in Figure 3F. Our model considers two principal components describing the activities of an “Activator (A)” and an “Inhibitor (I)”, both undergoing spatial diffusion and reaction (describing the interactions between the components represented in Figure 3F). The Activator is defined as having a more restricted range of action than the Inhibitor. For protein ligands diffusing in the extracellular space of a tissue, these differential diffusion characteristics could arise, for example, from differential binding affinities for components of a tissue’s extracellular matrix and need not be defined merely by the size of the macromolecule [see ref. 3]; a similar mechanism was found to be responsible for generating the necessary variation in diffusion coefficients for experimentally observed Turing patterns in a chemical reaction [see ref. 4] In their generic form, we have

$\begin{matrix} \frac{\partial A}{\partial t}=D_{A}\nabla^{2}A+f(A,I) -d_{A}A \\ \frac{\partial I}{\partial t}=D_{I}\nabla^{2}I+g(A,I) -d_{I}I \end{matrix}$ (1)

In the above, *D_A_* and *D_I_* describe Activator and Inhibitor diffusion rates, *d_A_* and *d_I_* describe Activator and Inhibitor decay rates and the kinetic functions *f(A,I)* and *g(A,I)* describe the up-regulation and constitutive application of the Activator and Inhibitor according to the proposed governing network. Specifically, the paths (1)-(5) depicted in Figure 3F are incorporated into the model through the choices

$f(A,I) =\overbrace{\frac{p_{A}A}{1+k_{1}A}}^{(1)}\times\overbrace{\frac{1}{1+\gamma k_{2}(x)I/\left(1+k_{3}A\right)}}^{(2,3)}$ and $g(A,I)=\overbrace{\frac{p_{I }A^{2}}{1+k_{4}A^{2}}}^{(4)}+\overbrace{B_{I}+C_{I}}^{(5)}$ (2)

In the equation for $f(A,I)$ (Activator up-regulation), the first term on the right hand side (RHS) describes (1) the self-activation/autocatalysis of the Activator while the second term describes both (2) the inhibition by the Inhibitor and (3) inhibition of the Inhibitor by the Activator. This third component arises from (i) our findings that extant placodes are not suppressed by application of recombinant BMPs to developing skin, (ii) that placodes do not express the BMP target gene *SOSTDC1* and (iii) reports that nascent placodes express BMP inhibitors [see ref. 5]. In the equation for$g(A,I)$, the first term on the RHS describes (4) up-regulation of Inhibitor by Activator while the second term describes (5) constitutive production of the Inhibitor. Note that the precise architecture considered here is not of fundamental importance to the reported results; for example, similar behavior can be observed for kinetics in which the “inhibition of Inhibition” component is neglected (data not shown). The parameters $p_{A},p_{I},k_{1},k_{3},k_{4},\gamma,d_{A},d_{I},D_{A},D_{I}$and $B_{I}+C_{I}$ are all constant in space/time (see Table S3). $k_{2}(x)$, as described in detail below, spatially varies to describe differential sensitivity to Inhibitor in neck/body regions.

The aim here is to determine whether the basic scheme in Figure 3F can both reproduce realistic feather placode patterns as well as explain the effects observed upon exogenous stimulation and suppression of BMP signaling. The choices for the kinetic functions in (2) are motivated both by their phenomenological representation of the network in Figure 3F as well as their similarity to well-studied kinetic systems, particular those of Gierer-Meinhardt type. As such, it is reasonable to expect diffusion-driven/Turing-type pattern formation for suitable parameter values and in Table S3 we list the default (and nondimensional) reference parameter set to be employed throughout our simulations. As demonstrated in Figure S8A, Equations (1)-(2) with this set generates a feather placode type pattern when solved on a two-dimensional field representing the emerging skin. We note that neither the precise parameter set nor the specific kinetic functions are critical in generating the results here. Indeed, qualitatively similar behavior to the results reported here have been observed both with other choices for the kinetics and distinct parameter sets (data not shown).

Feather placode patterning is simulated as taking place within an effectively two-dimensional and rectangular field of dimensions $L_{PA}\times L_{LML}$ that represents a posterior-anterior by lateral-medio-lateral portion of the emerging dermis-epidermis. Along each of the field edges we assume zero gain/loss of material and apply zero-flux/ Neumann boundary conditions while initial conditions are set as small randomized perturbations of the uniform steady state. To test the hypothesis that differential sensitivity to the Inhibitor is responsible for the distinct pattern responses of neck and body, for the simulations presented in Figure 3 and Figure 4 we assume that the parameter $k_{2}(x)$ (sensitivity to the inhibition in pathway (3)) varies with position along the posterior-anterior axis. The impact of spatially-varying parameters in Activator-Inhibitor type systems has been studied theoretically [see ref.s 6,7,8], where controlled heterogeneity was shown to generate a wide range of complex and spatially restricted patterning. Motivated by the sharp body to neck rise in BMP-sensitivity revealed by *SOSTDC1* expression (Figure 3E), we assume variation of the form

$k_{2}(x) =\frac{k_{NECK}+k_{BODY}}{2}+\frac{k_{NECK}-k_{BODY}}{2}\tanh(\alpha(x-L_{PA}/2))$. (3)

In the above, $x$represents the position along the posterior-anterior (body-neck) axis, $L_{PA}/2$represents the (approximate) boundary between neck and body, $k_{NECK}$and $k_{BODY}$ represent the sensitivities in the neck/body regions, and $\alpha$represents the gradient of the sensitivity slope. $\alpha$ was determined using the Image-Pro Plus software package to measure the gradient of the *SOSTDC1* in situ hybridization signal from neck to body on skin treated with 80 ng/ml recombinant Bmp12. For suitable parameters (Table S3) this generates a smooth slope consistent with the *SOSTDC1* gradient in BMP12 treated skin cultures. To recreate the exogenous BMP12 experiments, we considered stepwise increases to the constitutive Inhibitor parameter, $C_{I}$.

**Sensitivity analysis**

The model (1)-(3) generates patterning consistent with the exogenous application of Inhibitor, as illustrated in Figure 3. To further explore the impact of different manipulations we have applied a sensitivity analysis in which numerical simulations are performed following step-wise perturbations to the model parameters listed in Table S3 (note that for the sensitivity analysis we set $k_{2}=k_{BODY}$across the patterning field). For each perturbation the final pattern is compared against that produced by the default parameter set using the placode/Activator foci density as the quantitative indicator for equivalency. Patterns in which the placode density deviates by < 15% from the default set are classified as normal (e.g. Figure S8A and S8B). Patterns in which the placode density is significantly decreased or increased are indicated, see Figure S8C and S8D for representative examples. Certain perturbations shift the dynamics outside the parameter space in which Turing patterns are expected and no patterning occurs (i.e. uniformly distributed Activator/Inhibitor levels, see Figure S8G and S8H) while other perturbations transform the pattern of regularly sized foci/spots of high Activator level to fused foci and/or stripes (Figure S8E and S8F). In either such case, we indicate this loss of the regular pattern in Table S4.

Our sensitivity analysis demonstrates that specific parameter perturbations can dramatically alter the form of the emerging feather bud pattern and points to experimentally testable predictions. Focussing on the key parameter$k_{2}$, we note that decreases result in the gradual transition from a normal pattern to a striped pattern (e.g. Figure S8F) and finally to ubiquitously high Activator levels. Reduction of this parameter can be interpreted as a suppression of the activity of the Inhibitor (or suppression of its inhibitory action on the Activator). To determine the impact of performing this action on the full neck-body patterning field, we have performed *in silico* experiments in which the inhibitory action is steadily reduced through step-wise reductions of the scaling parameter ($\gamma$) on a field in which $k_{2}$is given by Equation (3). Our simulations (Figure 4) predict that fusions first take place in the body region before spreading to the neck region as this parameter is reduced. Note that this behaviour has also been observed under other parameter sets (data not shown).

**Numerical Method**

The numerical method invokes a Method of Lines scheme in which the spatial domain is discretized into a rectangular lattice (with fixed lattice size). Diffusion terms are approximated with a standard central differencing scheme and the subsequent system of differential equations is solved with the stiff-system integrator ROWMAP [ref. 9]. To test the accuracy of simulations, comparison simulations have been performed with various mesh size and error tolerances. A further control of the accuracy of the simulations was performed by comparing the results with those generated with an alternative numerical scheme based on the Alternating-Direction-Implicit (ADI) method.

1. Turing AM (1952) The Chemical Basis of Morphogenesis. Philosophical Transactions of the Royal Society of London Series B-Biological Sciences 237: 37-72

2. Gierer A, Meinhard H (1972) Theory of Biological Pattern Formation. Kybernetik 12: 30-39.

3. Hunding A, Sørensen PG (1988) Size adaptation of Turing prepatterns. J Math Biol 26: 27-39.

4. Lengyel I, Epstein IR (1991) Modeling of Turing Structures in the Chlorite-Iodide-Malonic Acid-Starch Reaction System. Science 251: 650-652.

5. Patel K, Makarenkova H, Jung HS (1999) The role of long range, local and direct signalling molecules during chick feather bud development involving the BMPs, follistatin and the Eph receptor tyrosine kinase Eph-A4. Mech Dev 86: 51-62.

6. Bensen DL, Sherratt JA, Maini PK (1993) Diffusion driven instability in an inhomogeneous domain. Bull Math Biol 55: 365-384.

7. Page K, Maini PK, Monk NAM (2003) Pattern formation in spatially heterogeneous Turing reaction-diffusion models. Physica D 181: 80-101.

8. Page K, Maini PK, Monk NAM (2005) Complex pattern formation in reaction-diffusion models with spatially varying parameters. Physica D 202: 95-115.

9. Weiner R, Schmitt BA, Podhaisky H (1997) ROWMAP - a ROW-code with Krylov techniques for large stiff ODEs. App Num Math 25: 303-319.
